# Frataxins Emerge as New Players of the Intracellular Antioxidant Machinery

**DOI:** 10.3390/antiox10020315

**Published:** 2021-02-20

**Authors:** Ana Belén Uceda, Josefa Donoso, Juan Frau, Bartolomé Vilanova, Miquel Adrover

**Affiliations:** 1 Departament de Química, Institut Universitari d’Investigació en Ciències de la Salut (IUNICS), Universitat de les Illes Balears, Ctra. Valldemossa km 7.5, E-07122 Palma de Mallorca, Spain; ana.uceda@uib.es (A.B.U.); josefa.donoso@uib.es (J.D.); juan.frau@uib.es (J.F.); bartomeu.vilanova@uib.es (B.V.); 2 Institut d’Investigació Sanitària de les Illes Balears (IdISBa), E-07120 Palma de Mallorca, Spain

**Keywords:** frataxins, copper, iron, metal-catalyzed oxidation, α-synuclein, reactive oxygen species

## Abstract

Frataxin is a mitochondrial protein which deficiency causes Friedreich’s ataxia, a cardio-neurodegenerative disease. The lack of frataxin induces the dysregulation of mitochondrial iron homeostasis and oxidative stress, which finally causes the neuronal death. The mechanism through which frataxin regulates the oxidative stress balance is rather complex and poorly understood. While the absence of human (Hfra) and yeast (Yfh1) frataxins turn out cells sensitive to oxidative stress, this does not occur when the frataxin gene is knocked-out in *E. coli*. To better understand the biological roles of Hfra and Yfh1 as endogenous antioxidants, we have studied their ability to inhibit the formation of reactive oxygen species (ROS) from Cu^2+^- and Fe^3+^-catalyzed degradation of ascorbic acid. Both proteins drastically reduce the formation of ROS, and during this process they are not oxidized. In addition, we have also demonstrated that merely the presence of Yfh1 or Hfra is enough to protect a highly oxidation-prone protein such as α-synuclein. This unspecific intervention (without a direct binding) suggests that frataxins could act as a shield to prevent the oxidation of a broad set of intracellular proteins, and reinforces that idea that frataxin can be used to prevent neurological pathologies linked to an enhanced oxidative stress.

## 1. Introduction

Friedreich’s ataxia (FRDA) is a neurodegenerative disease which prevalence strongly varies between different countries and even within the same country (from 1:20.000 to 1:150.000) [1]. It is characterized by an unsteady posture, falling, and a progressive difficulty in walking due to an impaired ability to coordinate movements [2]. FRDA appears as a result of an abnormal expansion of the GAA trinucleotide repeat in the first intron of the nuclear *FXN* gene [3]. Usually, healthy persons have roughly 6 to 36 repeats, whereas FRDA patients have a number of repeats ranging from 600 to 900 [4]. This gene expansion leads to lower expression levels of frataxin, a small acidic protein (pI ~ 4.9) conserved from bacterial to mammals [5]. Frataxin is present in all human cells, although higher levels are found in the heart and in the spinal cord, mostly in their mitochondrial membrane [3].

Human frataxin is one of the 850 proteins encoded in the nucleus and then directed to the mitochondrial membrane ([6]). Although it is firstly synthesized as a precursor protein (1–210), frataxin is then cut to form the mature form (81–120) [7]. Its structure contains two different regions: an intrinsically disordered *N*-terminal tail (81–90), and a C-terminal region (91–210; Hfra) with a well-defined globular fold that comprises two α-helices packed against a central anti-parallel β-sheet [8] (Figure S1A). The *N*-terminal region is absent in prokaryotes and poorly conserved in eukaryotes [9], whereas the C-terminal region is highly conserved throughout species, indicating that most of their residues must be essential either for the folding or for the function (Figure S1B). In fact, the α-β-α structure of the C-terminal region of Hfra is almost identical in yeast and bacterial frataxins (Yfh1 and CyaY, respectively) (Figure S1A).

All frataxins hold a hydrophobic region located at the β-sheet stretch, but also a negatively charged region on helix 1 (Figure S2), responsible for their metal binding, which occur through the clustered Asp and Glu residues [10]. However, little differences between the conserved C-terminal sequences of CyaY, Yfh1 and Hfra (Figure S1B) affect to their binding affinities. CyaY and Yhf1 bind Fe^2+^ at protein:metal ratios of 1:2, and with a higher affinity than Hfra, which binds seven Fe^2+^ cations. Hfra-Fe^3+^ complex has a higher affinity than the Hfra-Fe^2+^ complex, although sharing the same stoichiometry. A 1:7 stoichiometry was also observed for the CyaY-Fe^3+^ complex, although this protein has also lower affinity regions that makes it capable to bind up to 25 Fe^3+^ cations. On the other hand, Yfh1 is able to form a 1:1 complex with Fe^3+^, but its affinity is lower than that displayed by the Hfra-Fe^3+^ and Yfh1-Fe^2+^ complexes. In addition to iron, frataxins can also bind other cations such as Mn^2+^, Cu^+^, Cu^2+^ [11], or even Co^2+^ or Eu^3+^ [12]. In fact, the Yfh1-Cu^+^/Cu^2+^ affinities are higher than those shown by Yfh1 towards the Fe^2+/^Fe^3+^ cations (Table S1).

The affinity between frataxins and iron has let to consider them as iron binding proteins. Indeed, most of their biological roles are related with the iron metabolism [13], and as a result, frataxin deficiency causes a strong intracellular iron dysregulation [14].

Frataxin was initially suggested to keep iron in a bio-available form since ferritin could partially complement its lack [15]. This hypothesis was supported by the formation of iron-induced Yfh1 oligomers, able to transfer iron to other proteins [16]. Nevertheless, the formation of these oligomers have not been detected for Hfra [4].

In addition, Yfh1 seemed to be also involved in the heme biosynthesis [17] through a direct interaction with ferrochelatase [18]. However, healthy and FRDA patients did not show differences in the heme synthesis levels [19], although Hfra is able to interact with ferrochelatase in the presence of iron [20].

It was also observed that the Yfh1 deficiency decreased the activity of the Fe-S cluster-containing enzymes [21,22], which let to suggest that frataxins are also involved in the biosynthesis of the Fe-S clusters. The Fe-S clusters are formed on ISCU, a scaffold protein that takes persulfide from the NFS1 cysteine desulfurase, and electrons from the ferrodoxin FDX2, which reduce persulfide into sulfide. Yfh1 and Hfra bind the complex assembly [23], acting either as iron donors for the cluster assembly and/or as regulators of the entry of iron to ISCU [13]. Recently, the involvement of frataxin in the Fe-S cluster assembly has been clarified even more, as it has been proved that Hfra accelerates the persulfide transfer [24,25] while CyaY acts as an inhibitor of this process [13].

An equally relevant but yet less explored function of frataxin is its role as part of the mitochondrial machinery against oxidative stress. FDRA patients accumulate intracellular iron-protein deposits, but likely other metal-containing precipitates as the levels of zinc, copper, manganese, or aluminum were abnormally increased in a *Drosophila* model of FRDA [26]. These deposits induce the formation of reactive oxygen species (ROS) and trigger the oxidative stress [27,28], a key factor for the development of the neurodegeneration linked to FDRA. These ROS might also be responsible for the DNA damage linked to FRDA [29], which repair is also hampered by the lack of frataxin [30].

Therefore, it seems clear that frataxin must have a crucial role as protector against the mitochondrial oxidative stress. This plausible mechanism of action is supported by the increased cell resistance to oxidative stress and to iron accumulation linked to frataxin overexpression [31]. However, the mechanism through which frataxin displays this protective role is not fully understood yet. ROS accumulation in yeast, *Drosophila*, mice, and FRDA cells [28,32] let to suggest that frataxin deficiency might induce a deregulation of the cellular antioxidant defenses. In fact, frataxin deficiency silences the activation of the transcription factor Nrf2 [33,34], responsible for regulating several antioxidant genes in neurons [35]. Differently to healthy controls, superoxide dismutases (SODs) are not upregulated in FRDA patients treated with low doses of H_2_O_2_ and iron [36]. Moreover, the activities of SODs are notably enhanced upon interaction with Yfh1, which confirms the participation of this frataxin orthologue in the defense against oxidative stress [37]. In addition, frataxin deficiency decreases the total glutathione levels [32], while depleting the aconitase activity and disrupting the mitochondrial Fe-S cluster biogenesis [38].

Although it is clear that frataxin participates in the fine tuning of the cellular antioxidant machinery, it has not been investigated if frataxins are, per se, an active part of it. Evidence suggests their direct participation, which must be directly related to their metal-chelating ability (Table S1). Yfh1 reduces the formation of ROS from a Fe^2+^/H_2_O_2_ reaction mixture [39], whereas CyaY can attenuate the production of OH^●^ radicals [40]. However, the knock-out of the FXN homolog gene in *E. coli*, did not affect the cellular sensitivity to oxidants, which proves that CyaY must not be directly involved in the cellular protection against oxidative stress [41]. This is not the case of Hfra or Yfh1, whose absence confers cellular sensitivity to oxidative stress [27,31]. In addition, it has also been shown that extramitochondrial frataxin regulates the cytoplasmic oxidative balance [42]. Moreover, a Tat-fused Hfra, capable to penetrate the blood-brain barrier, is able to protect the dopaminergic neurons against oxidative stress [43] and also restore the activity of frataxin-depleted neurons [44].

Here, we aim to better understand the molecular mechanism through which Hfra and Yfh1 directly act against the oxidative stress. Therefore, we have studied their ability to inhibit ROS formation from Fe^3+^- and Cu^2+^- catalyzed ascorbic acid (AA) degradation, and their potential to protect other cytoplasmatic oxidation-prone proteins (e.g., α-synuclein, used here as a model). Our results unequivocally prove that both proteins have powerful antioxidant features, at the same time that act as potent shields to unspecifically prevent the oxidation of other proteins. Consequently, and for the first time, our findings point to frataxin as a main player of the antioxidant intracellular machinery.

## 2. Materials and Methods

### 2.1. Chemicals and Reagents

All chemicals and reagents were analytical grade and they were purchased either from Sigma-Aldrich (St. Louis, MO, USA) or from Acros Organics (Geel, Belgium). Moreover, all of them were used as received without further purification. All solutions used in this study were prepared by using milli-Q water.

### 2.2. Yeast Frataxin (Yfh1) Expression and Purification

Recombinant mature yeast frataxin from *Saccharomyces cerevisiae* (a 123-amino acid protein that included an additional *N*-terminal Met; Yfh1) was produced as described elsewhere [45]. Yfh1 was expressed in *E. coli* BL21(DE3) cells. These transformed cells were grown at 37 °C in a sterilized Luria Bertani media (LB) (25 g/L) containing ampicillin (100 μg/mL) while shaking at 180 rpm. At OD_600nm_ ~ 0.6, Yfh1 expression was induced with isopropyl-*β*-D-1-thiogalactopyranoside (IPTG) (1 mM) and further incubated for 6 h at 37 °C and 180 rpm. Afterwards, cells were harvested by centrifugation and re-suspended in lysis buffer (10 mM Tris-HCl, 1 mM EDTA, 1 mM PMSF, pH 8.0) and lysed by five cycles of sonication to liberate the protein and disrupt the DNA. Then, the suspension was centrifuged (at 4 °C and 4000 rpm during 30 min) and the soluble overexpressed protein was purified by two (NH_4_)_2_SO_4_ precipitation steps with a 40% cut to eliminate undesirable proteins, and a 65% cut to precipitate Yfh1. After this precipitation step, Yfh1 was subjected to an anion exchange chromatography using a Pharmacia Q-Sepharose column with a gradient to 1 M NaCl (the protein eluted at ~600 mM NaCl). The final purification step included a Pharmacia phenyl-sepharose column with a decreasing 1 M (NH_4_)_2_SO_4_ gradient (the protein eluted at ~800 mM (NH_4_)_2_SO_4_). The purified protein was then dialyzed into the desired buffer and stored at −25 °C until used. The purity of the obtained Yfh1 was checked using MALDI-TOF/TOF and SDS-PAGE electrophoresis (>95%). The concentration of Yfh1 was measured by UV–vis spectroscopy using a molar extinction coefficient estimated on the basis of its sequence: ε_Yfh1_280nm_ = 15,470 M^−1^·cm^−1^.

### 2.3. Human Frataxin (Hfra) Expression and Purification

Since full-length mature human frataxin tends to spontaneously degrade in vitro towards smaller fragments [8], we decided to study its evolutionary conserved stretch (91–210). In any case, the *N*-terminal tail of human frataxin (81–90) is poorly conserved (Figure S1B), thus the functional region must be located at the C-terminal region (91–210; here named as Hfra).

The C-terminal domain of human frataxin (91–210; Hfra) was expressed in *E. coli* BL21(DE3) as fusion protein with a His-tagged glutathione *S*-transferase (GST) and a cleave site for PreScission protease, as described previously [8]. The transformed cells were grown in sterilized LB (25 g/L) containing ampicillin (100 μg/mL) at 37 °C and 180 rpm. At OD_600nm_ ~ 0.6, Hfra expression was induced with IPTG (0.2 mM) and further incubated for 6 h at 37 °C and 180 rpm. Afterwards, cells were harvested by centrifugation and re-suspended in lysis buffer by five cycles of sonication. Then, a Ni-NTA chromatography was applied using a nickel column and the protein was eluted with a Tris buffered solution (20 mM; pH 8.0) containing 0.5 M imidazol. Purified Hfra was obtained in a subsequent step using gel filtration chromatography on a Superdex-75 HR 10/300 column. Finally, the His-tag was removed using the PreScission protease according to the manufacturer protocol (GE27-0843-01; Merck, Darmstadt, Germany) and then, the purified protein was dialyzed into the desired buffer and stored at −25 °C until used. The purity of Hfra was checked using MALDI-TOF/TOF and SDS-PAGE electrophoresis (>95%). The concentration of Hfra was measured by UV–vis spectroscopy using a molar extinction coefficient estimated on the basis of its sequence: ε_Hfra_280 nm_ = 26,930 M^−1^·cm^−1^.

### 2.4. Human α-Synuclein (α-syn) Expression and Purification

Recombinant human α-synuclein (α-syn) was produced as we described before [46]. In brief, *E. coli* BL21(DE3) transformed cells were grown in sterilized LB (25 g/L) containing ampicillin (100 μg/mL) at 37 °C and 180 rpm. At OD_600nm_ = 0.6–0.8 α-syn expression was induced with IPTG (1 mM) and further incubated during 4 h at 37 °C and 180 rpm. Afterwards, cells were centrifuged and the resulting pellet was resuspended in lysis buffer (10 mM Tris-HCl, 1 mM EDTA, 1 mM PMSF, pH 8.0) and stirred for 1 h at 4 °C. Cells were then lysed and the cellular debris were removed by centrifugation. Nucleic acids were removed by adding streptomycin sulfate (1% *w*/*v*) and stirring for 1 h at 4 °C, followed by centrifugation. The supernatant was supplied by the addition of (NH_4_)_2_SO_4_ (up to 0.295 g/mL) and additionally stirred for 1 h at 4 °C. The obtained pellet was collected by centrifugation, dissolved in 10 mM Tris-HCl (pH 7.4) and filtered through a 0.22 μm filter. The obtained solution was loaded onto an anion exchange column (GE Healthcare RESOURCETM Q; 6 mL) and α-syn was eluted with a NaCl gradient (0–600 mM). The purified protein was dialyzed into the desired buffer and stored at −25 °C until used. The purity of the obtained α-syn was checked using MALDI-TOF/TOF and SDS-PAGE electrophoresis (> 95%). α-Syn concentration was measured by UV-Vis spectroscopy using a molar extinction coefficient estimated on the basis of its amino acid content: ε_α-syn_280nm_ = 5960 M^−1^·cm^−1^.

### 2.5. Ascorbic Acid Degradation

The degradation rate of AA (70 μM) was studied at different concentrations of a sodium phosphate buffer (pH 7.4) (i.e., 5, 20, and 50 mM) that contained 150 mM NaCl. In addition, the AA degradation rate was studied in a 10 mM phosphate buffer (pH 7.4) containing different concentrations of NaCl (i.e., 0, 50, 100, 150, and 200 mM).

The degradation rate of AA (70 μM) was also studied in 10 mM phosphate buffer (pH 7.4) containing 150 mM of NaCl (*I* = 172.4 mM*;* from now named as buffer B1). The AA was alone or in the presence of 2.5 μM of Cu^2+^ or 2.5 μM of Fe^3+^. These reactions were also studied in the presence of ~5 μM EDTA. The degradation rate of AA (70 μM) in the presence of 2.5 μM of Cu^2+^ was also studied under anaerobic conditions. To carry out this experiment, aliquots of buffer B1 containing Cu^2+^ were subjected to N_2_ gas bubbling during 15 min. Afterwards, the AA was added and immediately, the AA degradation rate measured. The concentration of molecular oxygen in solution was measured in triplicate (for the studies carried out under aerobic but also anaerobic conditions) using a fiber-optic oxygen meter FireStingO2/FSO2-4 (PyroScience GmbH, Aachen, Germany) coupled to an oxygen probe (OXROB10).

All the reaction mixtures were prepared taking aliquots from an AA stock solution (3 mM) prepared in milli-Q water and stored in the dark at 4 °C during less than two days. In addition, we also used aliquots taken from stock solutions containing EDTA-Na_2_ (100 μM) prepared in buffer B1; CuCl_2_ or FeCl_3_ (100 μM) prepared in milli-Q water from more concentrated solutions (20 mM CuCl_2_ or FeCl_3_ in 40 mM glycine, a weak metal chelator used to avoid the formation of insoluble hydroxyls when adding the metal cations to the buffer B1 [47].

The degradation rate of AA (70 μM) in buffer B1 was also studied in the presence of Hfra (10 μM) or Yfh1 (at 0.5, 1 and 2.5 μM). In these assays, the protein was alone or in the co-presence of Cu^2+^ (2.5 μM) or Fe^3+^ (2.5 μM).

The temporal changes in the AA concentration were measured at 256 nm during 150 min, using a 1 cm quart cell and a Shimadzu UV-2401-PC double beam spectrophotometer (Shimadzu Europa GmbH, Duisburg, Germany). The absorbance of the buffer B1 was subtracted from the measurements. All the experiments were run in triplicate at 25 °C.

### 2.6. Dynamic Light Scattering (DLS) Measurements

DLS measurements of protein size distributions were performed using a Zetasizer Nano instrument (Malvern Instruments, Malvern, UK). The experiments were run at 90° scattering angle and using a laser with a wavelength of 633 nm. Stock solutions containing CuCl_2_ (1.2 mM) or FeCl_3_ (1.2 mM) were prepared in buffer B1. These solutions were prepared from more concentrated solutions (10 mM CuCl_2_ or FeCl_3_ in 20 mM glycine). Aliquots (100 µL) from samples containing Hfra (60 µM) alone or in the presence of Cu^2+^ or Fe^3+^ (600 µM) that were prepared in buffer B1, were used for DLS measurements at 25 °C. As soon as the metal cation was added to the Hfra solution, the sample was mixed and transferred to the DLS cuvette to start the measurement. Each measurement included the accumulation of 30 different correlation curves.

### 2.7. Superoxide Anion (O_2_^●−^) Formation

Nitroblue tetrazolium (NBT) was used to monitor the time-dependent formation of O_2_^●−^ during the Fe^3+^-catalyzed AA degradation. Once O_2_^●−^ is formed, it rapidly reacts with NBT to yield formazan, which increases the absorbance of the overall solution at 560 nm [48,49]. A reaction mixture containing AA (70 μM), Fe^3+^ (2.5 μM) and NBT (50 μM) was prepared in 10 mM sodium phosphate buffer at pH 7.4. The UV–vis spectra of this reaction mixture were recorded at different incubation times (at 25 °C) using a 1 cm plastic cell and a Shimadzu UV-2401-PC double beam spectrophotometer (Shimadzu Europa GmbH, Duisburg, Germany). The absorbance of the buffer was subtracted from the measurements. Experiments were run in duplicate.

### 2.8. Hydrogen Peroxide (H_2_O_2_) Formation

The effect of Yfh1 and Hfra on the H_2_O_2_ formation was studied using the red peroxidase kit assay (MAK165; Sigma-Aldrich, St. Louis, MO, USA). Red peroxidase substrate (RS) is a non-fluorescent compound that reacts with H_2_O_2_ (1:1) in the presence of horseradish peroxidase (HRP) to form the fluorescent resorufin. RS was reconstituted with 250 μL of DMSO, whereas HRP was reconstituted with 1 mL of the assay buffer (20 U/mL). A solution containing 50 μL of the RS stock solution, 200 μL of the HRP stock solution, and 4.75 mL of the assay buffer was prepared (master mix; MX). The time-dependent H_2_O_2_ formation was measured on 50 μL of samples containing AA (70 μM) and Cu^2+^ (2.5 μM) or Fe^3+^ (2.5 μM), in the absence or in the presence of Hfra and Yfh1 at 0.5, 2.5, and 10 μM concentrations. In all preparations, AA was the final reagent added before starting the fluorescence measurements, which were recorded during 150 min. Assay buffer was used as background fluorescence and subtracted from the data. Control experiments without Cu^2+^ or Fe^3+^, and without AA were also done. The concentrations of H_2_O_2_ in the reaction mixtures were determined from a standard calibration curve, obtained using freshly prepared H_2_O_2_ solutions with concentrations of 0, 0.5, 2, 4, 10, 20, 30, and 50 μM (prepared from a 20 mM H_2_O_2_ stock solution). 50 μL of the different H_2_O_2_ stock solutions were mixed with 50 μL of the MX solution, incubated 30 min at 25 °C and afterwards, their fluorescence intensities were recorded. All fluorescence measurements were done in triplicate at 25 °C on a Varian Cary Eclipse fluorescence spectrophotometer (Agilent, Santa Clara, CA, USA) using 96-well plates (λ_exc_ 540 nm; λ_em_ 590 nm).

### 2.9. Total Free Radical Formation from Cu^2+^- and Fe^3+^-Catalyzed AA Degradation

A fluorescein stock solution (2 mM) was prepared in buffer B1 and added to a final concentration of 26 μM to reaction mixtures containing AA (70 μM) alone or in the presence of 2.5 μM Cu^2+^ or Fe^3+^, which were prepared in buffer B1. These mixtures were also studied in the presence of 0.5 or 10 μM Hfra and Yfh1. The temporal variation of the fluorescence signal at 518 nm (λ_exc_ 490 nm) was followed during 150 min. All the experiments were run in duplicated at 25 °C using a Varian Cary Eclipse fluorescence spectrophotometer and quartz cells of 1 cm path length.

### 2.10. Analysis of the Formation of the Hydroxyl Radical (HO^●^)

The effect of Hfra and Yfh1 on the formation of HO^●^ was studied using the coumarin-3-carboxylic acid (3-CCA). In the presence of HO^●^, 3-CCA is rapidly oxidized to the fluorescent 7-hydroxy-coumarin-3-carboxylic acid (7-OH-CCA) (λ_max_exc_ 395 nm; λ_max_em_ 450 nm), thus allowing to record the time-dependent HO^●^ formation [50]. A 3-CCA stock solution (20 mM) was prepared in 20 mM sodium phosphate buffer (pH 9.0) and added to a final concentration of 100 μM to reaction mixtures that always contained 70 μM AA and 2.5 μM Cu^2+^ or Fe^3+^. The AA/Cu^2+^ reaction mixtures were also prepared in the presence of 10 μM Hfra or 0.5 μM Yfh1. Solutions containing 70 μM AA or 2.5 μM Cu^2+^ alone were used as controls. The temporal variation of the fluorescence signal at 450 nm was followed during 150 min. All the experiments were run in triplicate or higher at 25 °C using a Varian Cary Eclipse fluorescence spectrophotometer (Agilent, Santa Clara, CA, USA) and quartz cells of 1 cm path length.

### 2.11. MALDI-TOF/TOF MS Study of the Effect of Reactive Oxygen Species (ROS) on the Molecular Weight of Frataxins and α-Synyclein

MALDI-TOF/TOF studies were performed on a Bruker Autoflex III MALDI-TOF/TOF spectrometer (Bruker, Billerica, MA, USA) equipped with a 200-MHz smart-beam pulsed N2 laser (λ 337 nm). Aliquots of 1 μL of mixtures containing 10 μM or 5 μM protein (Hfra, Yfh1 and/or α-syn), 70 μM AA and 2.5 μM Cu^2+^ or Fe^3+^ prepared in buffer B1, were taken after 0 and 150 min of incubation (25 °C) and supplemented by trifluoroacetic acid (TFA) (0.2% *v/v*). Samples were then combined with 1 μL of matrix solution (10 μg of sinapinic acid in a solution water:acetonitrile (70:30) containing 0.1% TFA), and a 0.5 μL aliquot of this mixture was spotted onto a steel target plate (MTP 384), air-dried and subjected to mass determination. The IS1 and IS2 voltages were 20 kV and 18.5 kV respectively, and the lens voltage was 7.5 kV. Measurements were performed using a positive reflector mode with matrix suppression below 400 Da. The spectra were calibrated externally using a protein calibration standard (3600–17,000 Da) from Bruker. The experiments were performed in duplicate.

### 2.12. Fluorescence Study of the Di-Tyrosine Formation

The Tyr-Tyr crosslinking was studied on reaction mixtures containing 70 μM of AA, 2.5 μM of Cu^2+^ or Fe^3+^, and 10 μM of Hfra or Yfh1. These reaction mixtures were prepared in buffer B1 and incubated at 25 °C during 150 min. The formation of di-Tyr involves the loss of the Tyr fluorescence signal (λ_max_em_ ~ 305 nm) concomitant with an increase in the fluorescence emission intensity between 405–410 nm [51]. Consequently, the fluorescent spectra of the different reaction mixtures were acquired between 375 and 525 nm (λ_exc_ 325 nm) before and after incubation using a Varian Cary Eclipse fluorescence spectrophotometer and quartz cells of 1 cm path length. Experiments were performed in duplicate.

### 2.13. Scavenging Capacity of Hfra, Yfh1, and α-Syn on the HO^●^ Radical

The cupric reducing antioxidant capacity (CUPRAC) method was applied to analyze the ability of the different proteins to trap the HO^●^ radical [52,53]. CUPRAC method involves the formation of HO^●^ as a result of the reaction between Fe^2+^ and H_2_O_2_. After this reaction is completed, the latter is degraded using catalase to avoid chemical interferences during the determination. HO^●^ can hydroxylate salicylic acid, which is able to reduce the neocuproine-Cu^2+^ to neocuproine-Cu^+^ (yellow; λ_abs_max_ 450 nm). If the different proteins are able to react with HO^●^, this would avoid the hydroxylation of salicylic acid and consequently, the formation of the UV-active neocuproine-Cu^+^ complex.

For this study, stock solutions of salicylic acid (10 mM), FeCl_2_ (20 mM in milli-Q water containing 40 μL of HCl 1M for each ml of solution), EDTA (20 mM), H_2_O_2_ (10 mM), CuCl_2_ (10 mM), and AcNH_4_ (1 M) were prepared in milli-Q water. Additionally, neocuproine (7.5 mM) was prepared in ethanol, whereas a catalase solution (298 U/mL) was prepared in buffer B1. All stock solutions were stored at 4 °C in dark until use. Initially, reaction mixtures containing 0.5 mM salicylic acid, 0.5 mM Fe^2+^, 0.5 mM EDTA, and 0.5 mM H_2_O_2_, in the absence or in the presence of α-syn (10 μM), Hfra (10 μM), Yfh1 (10 μM), α-syn (10 μM) + Hfra (10 μM) or α-syn (10 μM) + Yfh1 (10 μM), were prepared in the buffer B1 and incubated during 10 min at 37 °C and at 500 rpm. Afterwards, catalase was added to a final concentration of 15 U/mL and further incubated during 30 min under the same conditions. Later on, 100 μL of the resulting mixtures were diluted in 1mL of milli-Q water containing 1 mM of Cu^2+^, 0.75 mM of neocuproine and 0.2 M of AcNH_4_. These solutions were incubated during 2.5 h at room temperature and their UV–vis spectrum were recorded at 25 °C using a 1 cm quartz cell and a Shimadzu UV-2401-PC double beam spectrophotometer (Shimadzu Europa GmbH, Duisburg, Germany). The absorbance of a 1 mL mixture containing 1 μM of Cu^2+^, 0.75 μM of neocuproine, 0.2 M of AcNH_4_ and 100 μL of milli-Q water was subtracted from the measurements. Experiments were run in duplicate.

### 2.14. Study of the Interactions between Hfra and Yfh1 with α-Syn

The bindings of Hfra and Yfh1 with α-syn were studied by chemical cross-linking experiments, carried out using the ethylene glycol bis-succinimidyl succinate (EGS). Reaction mixtures prepared in buffer B1 contained: (i) 10 μM α-syn; (ii) 10 μM Hfra: (iii) 10 μM Yfh1: (iv) 10 μM α-syn and 10 μM Hfra; and (v) 10 μM α-syn and 10 μM Yfh1. These mixtures were incubated in the absence and in the presence of 0.1 mM EGS during 30 min at 25 °C. Afterwards, the reactions containing EGS were quenched by the addition of 50 mM Tris (pH 7.5) and further incubated during 15 min at 25 °C. The obtained samples were analyzed using SDS-PAGE electrophoresis and stained using silver staining. Additionally, all the reaction mixtures were analyzed by MALDI-TOF/TOF using the experimental approach already described before.

### 2.15. Sodium Dodecyl Sulfate-Polyacrylamide Gel Electrophoresis

Sodium dodecyl sulfate-polyacrylamide gel electrophoresis (SDS-PAGE) studies carried out in this work were performed by taking 15 µL of the different samples that were analyzed and mixed with 15 µL of Laemmli sample buffer (Bio-Rad, Hercules, CA, USA). The resulting mixture was then loaded onto 4–20% Mini-Protean TGX precast gels (Bio-Rad, Hercules, CA, USA). The gels were subjected to a voltage of 200 V for 45 min at room temperature using running buffer (100 mM Tris, 100 mM glycine and 0.1% SDS). Proteins were visualized with silver staining (Thermo Scientific, Waltham, MA, USA).

## 3. Results and Discussion

### 3.1. Degradation Rate of Ascorbic Acid Depends on the Concentrations of Phosphate and NaCl

FDRA patients have an unbalance in the iron homeostasis [26,54] and an expanded intracellular cooper distribution [55]. This unavoidably trigger an increase of the free radical release and therefore, an enhanced oxidative stress, directly linked to the pathogenesis of FRDA [28]. These findings let us to suggest a rather trivial but yet unproven hypothesis: the metal-chelating ability of frataxins could also be part of the cellular machinery against oxidative stress.

**Figure 1:**
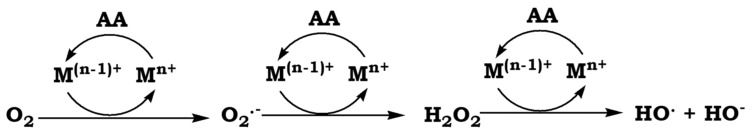
Reaction cycle for the production of ROS from molecular oxygen and AA [60]. M^n+^ catalyzes the oxidation of AA to dehydroascorbic acid through the formation of a M^n+^-AA complex, which binds to O_2_. In the complex, M^n+^ mediates the electron transfer from AA to the O_2_ in a series of reactions that leads to the production of superoxide anion radical (O_2_^●−^) and H_2_O_2_. In these reactions M^n+^ is reduced to M^(n−1)+^ and oxidized back in a Fenton-type of reaction, which constitutes the source of one electron equivalent required for the reduction of H_2_O_2_. The final product of these reactions is OH^●^.

To investigate this possibility, we took as reactions model the Cu^2+^- and Fe^3+^-catalyzed ascorbic acid (AA) degradations. AA is present at high concentrations in cells, especially in neurons (~10 mM), where it scavenges reactive oxygen species (ROS) [56,57]. However, if free Fe^3+^ and Cu^2+^ cations are present, they form complexes with AA, stimulating its oxidation while producing ROS (Figure 1) [58,59].

The metal-catalyzed oxidation rate of AA is highly dependent on the environmental conditions [61]. Hence, before to test the effect of Yfh1 and Hfra on this reaction, we screened different phosphate (pH 7.4) and NaCl concentrations (as we aimed to mimic the intracellular environment), to have the lowest non-catalyzed degradation rate of AA, which would diminish the relevance of this side-reaction. Its degradation rate decreased upon increasing the NaCl concentration (Figure S3A), likely due to the inhibitory effect of this salt on the O_2_-AA interaction [62]. On the contrary, phosphate seems to catalyze the AA oxidation (Figure S3B). In any case, the degradation rate of AA was always quicker than when EDTA was present, suggesting that even small metal traces might be present in solution (Figure S4A). As expected, the degradation rate of AA also depends on the concentration of molecular oxygen in the reaction mixture (8.1 ± 2 mg/L), as it drastically decreased under anaerobic conditions (Figure S4B).

From the obtained data, we decided to perform the protein containing studies using a 10 mM phosphate buffer supplied with 150 mM NaCl. These conditions were chosen because the reduction in the degradation rate observed when using 5 mM phosphate or 200 mM NaCl was not significant, and at the same time we better ensured a constant pH and a low protein aggregation rate (NaCl stimulates protein aggregation) [63].

### 3.2. Yfh1, but not Hfra, Inhibits the Cu^2+^- and Fe^3+^-Catalyzed Degradation of AA

Cu^2+^ catalyzed the oxidation of AA, and its catalytic effect was higher than that shown by Fe^3+^. Since this effect was completely abolished in presence of EDTA (Figure S4A), we assumed that metal-chelating proteins (e.g., Hfra or Yfh1) could act in the same way.

**Figure 2:**
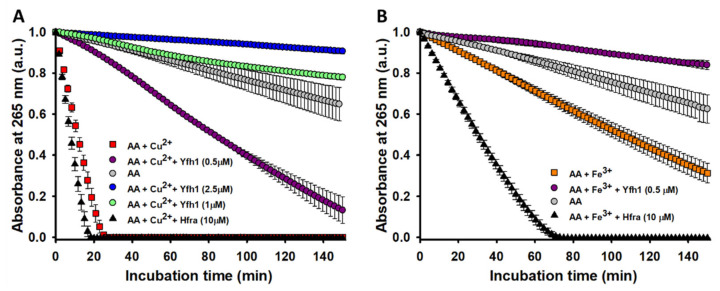
Effect of Hfra and Yfh1 on the Cu^2+^- and Fe^3+^-catalyzed degradation rate of AA. (**A**) Time-dependent AA (70 μM) degradation at 25 °C measured by the decrease in its absorbance at 265 nm when AA was alone (●), in presence of Cu^2+^ (2.5 μM) (■), in the presence of Cu^2+^ (2.5 μM) and Yfh1 (0.5 μM) (●), in the presence of Cu^2+^ (2.5 μM) and Yfh1 (1 μM) (●), in the presence of Cu^2+^ (2.5 μM) and Yfh1 (2.5 μM) (●), and in the presence of Cu^2+^ (2.5 μM) and Hfra (10 μM) (▲). (**B**) Time-dependent AA (70 μM) degradation at 25 °C measured by the decrease in its absorbance at 265 nm when AA was alone (●), in presence of Fe^3+^ (2.5 μM) (■), in the presence of Fe^3+^ (2.5 μM) and Yfh1 (0.5 μM) (●), and in the presence of Fe^3+^ (2.5 μM) and Hfra (10 μM) (▲). In both panels, the data points are the mean from all the replicas, and the error bars represent standard deviation from the different independent measurements.

In fact, Yfh1 totally inhibited the degradation of AA when Cu^2+^ was present (AA only degraded <0.5% after 150 min of incubation with 10 μM Yfh1; *data not shown*). Its inhibitory effect had a concentration-dependent behavior (Figure 2A), and even at a Yhf1:Cu^2+^ ratio of 1:5, the time needed to degrade the 50% of AA increased from 10.4 to 84.4 min. The Fe^3+^-catalyzed degradation of AA was also partially inhibited by Yfh1. The presence of Yfh1 at a 5-fold lower concentration than that of Fe^3+^, also enhanced the time to degrade the 50% of AA from 107 to 463 min (Figure 2B). The inhibitory effect of Yfh1 was also observed in the absence of Cu^2+^/Fe^3+^ (Figure S5), which suggest that Yfh1 can also chelate trace metals.

Hfra is able to bind Cu^2+^ with a slightly higher affinity than Yfh1 (Table S1). Hence, we expected a similar inhibitory effect. However, we observed a completely opposite trend. The time needed to degrade the 50% of AA in the presence of Cu^2+^ slightly decreased upon increasing the Hfra concentration. In fact, it decreased an ~40% in presence of 10 μM Hfra (Figure 2A). This effect was even more pronounced on the Fe^3+^-catalyzed degradation of AA, where the presence of 10 μM Hfra reduced this time in ~71% (Figure 2B).

Altogether, these results prove that the ability of frataxins to protect against the metal-catalyzed oxidation must depend on other factors different from their similar metal binding affinity. Yfh1 and Hfra have different propensities to form metal-induced assemblies [64]. Thus, we investigated if this could be related with their different mechanism of action on Cu^2+^/Fe^3+^-catalyzed AA oxidation. While Cu^2+^ does not induce the formation of Yfh1 oligomers [65], Fe^3+^ polymerizes Yfh1 by forming different multimers working as storage compartment for Fe^3+^, but also as part of the iron detoxification strategy [66]. On the other hand, Fe^3+^ also induces the formation of unstable Hfra^81–210^ oligomers [67]. However, there is not data reporting the metal-induced oligomeric propensity of the short Hfra construct (91–210). DLS experiments recorded on our Hfra^91–210^ prove that Cu^2+^ is able to induce the formation of protein assemblies (with a *Rh* of ~8 nm), but those are smaller than those formed in the presence of Fe^3+^. In fact, Fe^3+^ clumps Hfra into two different oligomeric forms, one with *Rh* ~ 0.7 μm and another with a *Rh* ~ 1.4 μm (Figure S6).

The obtained results reveal that Yfh1 and Hfra have different ability to protect AA (but also likely other cellular compounds prompt to be degraded through a metal-catalyzed mechanism). While Yfh1 acts as strong antioxidant, Hfra alone (Figure S5) but especially Hfra-metal complexes, seem to have an enhanced capacity to catalyze the AA degradation. This difference cannot be ascribed to a different metal binding ability (Yfh1 and Hfra have similar Fe^3+^ affinities) nor to a different metal-induced oligomeric propensity (Yhf1 and Hfra form oligomers in presence of Fe^3+^). Consequently, other sequentially/structural factors, or even differences in the geometries of the complexes, might be behind the differences observed between Yfh1 and Hfra. In any case, our data reveal that Yfh1, differently to Hfra, could hidden a new cellular function: act per se as a shield against the metal-catalyzed intracellular oxidations.

### 3.3. Effect of Hfra and Yfh1 on the Cu^2+^ and Fe^3+^-Catalyzed Formation of H_2_O_2_

Under aerobic conditions, metal cations mediate the electron transfers steps from AA to O_2_, which is reduced to firstly form H_2_O_2_ and finally H_2_O. During these processes, superoxide anion radical (O_2_^●−^) and hydroxyl radical (HO^●^) are formed as intermediates of the partial O_2_ reduction (Figure 1). If this occurs in vivo, both radicals but also H_2_O_2_, rapidly react with biomolecules causing the oxidative stress. Therefore, we also studied whether the effect of Yfh1 and Hfra on the degradation rate of AA would also have consequences on the formation of O_2_^●−^, H_2_O_2_, and HO^●^.

**Figure 3:**
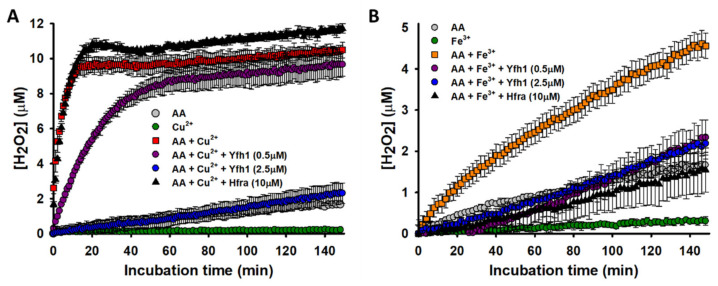
Effect of Hfra and Yfh1 on the formation of H_2_O_2_. (**A**) Time-dependent formation of H_2_O_2_ during the Cu^2+^-catalyzed oxidation of AA. The concentration of H_2_O_2_ was measured by the increase in resorufin fluorescence at 590 nm. The different reaction mixtures contained: (i) Cu^2+^ (2.5 μM) alone (●); (ii) AA (70 μM) alone (●); (iii) AA (70 μM) and Cu^2+^ (2.5 μM) (■); (iv) AA (70 μM), Cu^2+^ (2.5 μM) and Yfh1 (0.5 μM) (●); (v) AA (70 μM), Cu^2+^ (2.5 μM) and Yfh1 (2.5 μM) (●); and (vi) AA (70 μM), Cu^2+^ (2.5 μM) and Hfra (10 μM) (▲). (**B**) Time-dependent formation of H_2_O_2_ during the Fe^3+^-catalyzed oxidation of AA. The reaction mixtures contained: (i) Fe^3+^ (2.5 μM) alone (●); (ii) AA (70 μM) alone (●); (iii) AA (70 μM) and Fe^3+^ (2.5 μM) (■); (iv) AA (70 μM), Fe^3+^ (2.5 μM) and Yfh1 (0.5 μM) (●); (v) AA (70 μM), Fe^3+^ (2.5 μM) and Yfh1 (2.5 μM) (●); and (vi) AA (70 μM), Cu^2+^ (2.5 μM) and Hfra (10 μM) (▲). In both panels, the data points are the mean from all experiments, and the error bars represent standard deviation from the different independent measurements.

As we proved earlier, once O_2_^●−^ is formed, it reacts faster with free AA (towards the formation of H_2_O_2_) than with NTB (the detection probe). Hence, the formation of free O_2_^●−^ was undetectable during the Cu^2+^-catalyzed AA degradation [46], nor during the Fe^3+^-catalyzed AA degradation (Figure S7). Consequently, we could not study the effect of Yfh1/Hfra on the formation of O_2_^●−^. If O_2_^●−^ takes an additional electron from AA, it evolves to H_2_O_2_. Both Fe^3+^ and Cu^2+^ are able to catalyze this process, although the latter has a higher catalytic efficiency (Figure 3). In fact, this correlates with their effectiveness to catalyze the AA oxidation (Figure 2).

Yfh1 was able to reduce in a concentration-dependent manner the rate of Cu^2+^-catalyzed formation of H_2_O_2_. In fact, when Yfh1 and Cu^2+^ were at 1:1 ratio, H_2_O_2_ was formed at a similar rate and yield than from the non-catalyzed degradation of AA (Figure 3A). Yfh1 also abolished the Fe^3+^-catalyzed formation of H_2_O_2_. However, it did not show a concentration-dependent effect, as even tiny amounts of Yfh1 (0.5 μM) decreased the H_2_O_2_ formation at levels comparable with those obtained from the non-catalyzed degradation of AA (Figure 3B). This proves that Yfh1 has a higher potential to inhibit H_2_O_2_ formation form Fe^3+^-catalyzed AA oxidation than from Cu^2+^-catalyzed AA oxidation.

As expected from the previously obtained data, the presence of Hfra slightly increased the H_2_O_2_ concentration formed from the Cu^2+^-catalyzed AA oxidation (Figure 3A). However, we surprisingly observed how Hfra was able to reduce the Fe^3+^-catalyzed formation rate of H_2_O_2_ at levels similar with those observed during the non-catalyzed oxidation of AA (Figure 3B).

Consequently, the obtained data prove that Yhf1 and Hfra have different roles on the formation of H_2_O_2_ from the metal-assisted O_2_^●−^ reduction. While Yfh1 is able to diminish the formation of H_2_O_2_ independently of the metal cation acting as a catalyzer, Hfra cannot inhibit the Cu^2+^-catalyzed H_2_O_2_ formation but it has a notably effect on the Fe^3+^-catalyzed process. These results let us to hypothesize that, although the Hfra-Fe^3+^ complexes accelerate the degradation rate of AA (Figure 2B), it is likely that they are also able to redirect the degradation mechanism towards the formation of other chemical species different than ROS.

### 3.4. Hfra and Yfh1 Inhibit the Formation of Free Radicals from Cu^2+^-Catalyzed Degradation of AA

**Figure 4:**
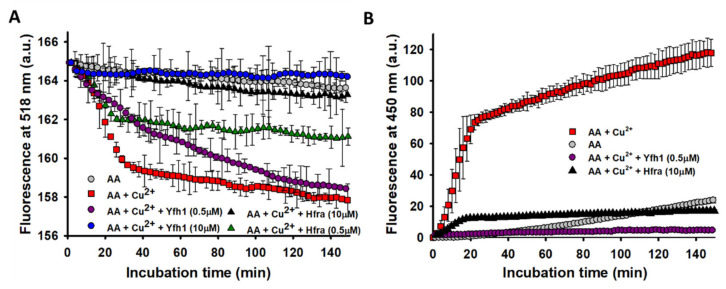
Effect Cu^2+^ on the overall free radical and hydroxyl radical (OH^●^) formation from the AA degradation. (**A**) Time-dependent overall free radical formation monitored by the decrease in the fluorescence intensity of fluorescein (26 μM; λ_exc_ 490 nm) of a solution prepared in buffer B1 that contained: (i) AA (70 μM) alone (●); (ii) AA (70 μM) and Cu^2+^ (2.5 μM) (■); (iii) AA (70 μM), Cu^2+^ (2.5 μM) and Yfh1 (0.5 μM) (●); (iv) AA (70 μM), Cu^2+^ (2.5 μM) and Yfh1 (2.5 μM) (●); v) AA (70 μM), Cu^2+^ (2.5 μM) and Hfra (10 μM) (▲); and vi) AA (70 μM), Cu^2+^ (2.5 μM) and Hfra (0.5 μM) (▲). The experimental data was smoothed using the negative exponential function in Sigmaplot. (**B**) Time-dependent formation of HO^●^ measured by the increase in the fluorescence of 3-CAA at 450 nm (λ_exc_ = 395 nm) of a solution prepared in buffer B1 that contained: (i) AA (70 μM) alone (●); (ii) AA (70 μM) and Cu^2+^ (2.5 μM) (■); (iii) AA (70 μM), Cu^2+^ (2.5 μM) and Yfh1 (0.5 μM) (●); and (iv) AA (70 μM), Cu^2+^ (2.5 μM) and Hfra (10 μM) (▲). In both panels, the data points are the mean from all experiments, and the error bars represent standard deviation from the different independent measurements.

If this hypothesis were true, it would be expected that the formation of the free radicals arising from the metal-catalyzed oxidation of AA would diminish in presence of frataxins. Thus, their formation from the Cu^2+^- and Fe^3+^-catalyzed AA degradation was monitored from the decrease in the fluorescence of fluorescein [68,69]. While the presence of Fe^3+^ did not increase the production of free radicals (Figure S8A), Cu^2+^ highly stimulated their formation (Figure 4A). This different catalytic behavior was already described for the formation of H_2_O_2_, and it can be ascribed to a high oxidizing capacity of the Fe^3+^/Fe^2+^ pair (*E*^0^ = 0.77 V) relative to the O_2_/H_2_O_2_ (*E*^0^ = 0.28 V) and the H_2_O_2_/HO^●^ (*E*^0^ = 0.38 V) pairs [70], which is not shown by the Cu^2+^/Cu^+^ pair (*E*^0^ = 0.15 V).

The presence of small amounts of Yfh1 (0.5 μM) slightly delayed the formation of free radicals, but it did not change their final amount. However, upon increasing its concentration, the amount of free radicals was reduced to the point that their formation was completely inhibited at 10 μM Yfh1. Regardless Hfra was not able to inhibit the formation of Cu^2+^-catalyzed H_2_O_2_, it exhibited a remarkable potential to inhibit its further reduction towards the formation of free radicals. Even its inhibitory potential was higher than that shown by Yfh1 (at 0.5 μM Yfh1 was only able to reduce the formation of free radicals in ~12.5%, while Hfra reduced it in ~50%) (Figure 4A).

Our data prove that Yfh1 can inhibit the Cu^2+^- and Fe^3+^-catalyzed degradation of AA, but also the formation of cell-damaging products generated during this process, such as H_2_O_2_ and free radicals. In contrast, Hfra accelerates the metal-catalyzed degradation of AA, as well as the Cu^2+^-catalyzed formation of H_2_O_2_, while inhibiting the Fe^3+^-catalyzed formation of H_2_O_2_. Nevertheless, Hfra showed a remarkable potential to inhibit the overall formation of free radicals, which might be biologically related to its antioxidant role.

### 3.5. Hfra and Yfh1 Inhibit the Formation of Hydroxyl Radical (HO^●^)

The effectiveness of frataxins to inhibit the formation of free radicals should be also reflected to its capacity to inhibit the Fe^3+^/Cu^2+^-catalyzed reduction of H_2_O_2_ towards the formation of HO^●^. As expected from the fluorescein assay, Fe^3+^ did not increased the formation of HO^●^ further than that formed from the non-catalyzed oxidation of AA (Figure S8B). However, the presence of Cu^2+^ directed the degradation of AA towards the formation of remarkable quantities of HO^●^ (Figure 4B). The formation of HO^●^ was completely inhibited in the presence of 0.5 μM Yfh1. Hence, the free radicals formed during the AA oxidation when Yfh1 (0.5 μM) was present (Figure 4A), must correspond to other radicals different than HO^●^. On the other hand, Hfra was able to delay the formation rate of HO^●^ during the first 20 min of incubation. After that, the HO^●^ concentration remained constant, but ~83% lower than that obtained in the absence of Hfra (Figure 4B).

Consequently, our data prove that Yfh1 is able to inhibit the free radical formation from Cu^2+^-catalyzed AA degradation. Hoverer, its mechanism of action is especially effective on the reduction of H_2_O_2_ to form HO^●^. In addition, we demonstrate that Hfra is able to inhibit the free radical formation and consequently, it can inhibit (at least in part) the reactions yielding HO^●^. Therefore, the direct intervention of frataxins on the reactions producing free radicals reinforce their role as part of the endogenous antioxidant machinery.

### 3.6. Frataxins Do Not Undergo Oxidation upon Incubation with AA and Fe^3+^/Cu^2+^

However, a complete comprehension of the antioxidant role of frataxins cannot be achieved without getting insights on their propensity to undergo chemical modifications concomitant to their intervention against oxidative stress. Hence, we used MALDI-TOF/TOF spectrometry to evaluate the oxidation level of Yfh1 and Hfra during the Cu^2+^/Fe^3+^-catalyzed oxidation of AA.

**Figure 5:**
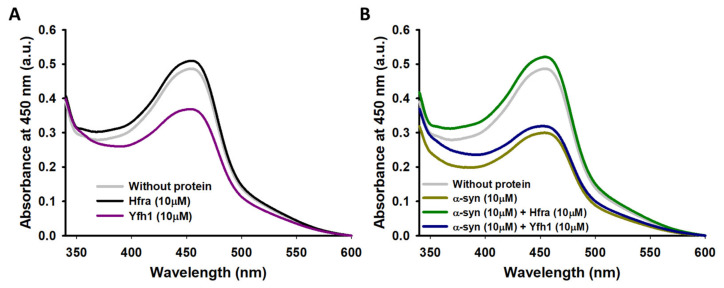
UV–vis spectra of the neocuproine-Cu^+^ complex formed from the hydroxylation of salicylic acid by HO^●^ in the absence (*grey*) (**A**,**B**) or in the presence of 10 μM Yfh1 (*purple*) (**A**), 10 μM Hfra (*black*) (**A**), 10 μM α-syn (*dark-yellow*) (**B**), 10 μM α-syn and 10 μM Yfh1 (*blue*) (**B**), and 10 μM α-syn and 10 μM Hfra (*green*). All the experiments were carried out in duplicate.

The molecular weight of Yfh1 did not change when it was incubated with Fe^3+^/AA, nor with Cu^2+^/AA (Figure S9A,B), which proves that these oxidizing conditions are not strong enough to covalently modify Yfh1. Moreover, di-Tyr crosslinks resulting from the radical coupling to Tyr residues [51,71] were not detected between any of the three different Tyr of Yfh1, as the fluorescence emission intensity between 405–410nm did not increase [51] (Figure S10). However, we observed that Yfh1 was able to partially trap chemically synthetized HO^●^ (Figure 5A), thus indicating that HO^●^ radicals would be able to damage Yfh1 in case they were formed. Altogether these results prove that, although Yfh1 is prompt to be oxidized by HO^●^, this does not occur under the conditions designed to mimic the cellular oxidative stress. In fact, this somehow self-protecting mechanism must be linked to its remarkable ability to inhibit the metal-catalyzed AA degradation and therefore, the formation of damaging ROS.

In like manner, the molecular weight of Hfra was not modified when it was incubated with Fe^3+^/AA, nor with Cu^2+^/AA (Figure S9C,D), thus proving that Hfra resist the chemical modifications that would induce the reaction products of the metal-catalyzed degradation of AA. In addition, any of its seven Tyr seemed to be cross-linked through the formation of di-Tyr (Figure S10). However, differently of what was observed for Yfh1, Hfra was not able to trap chemically synthetized HO^●^ (Figure 5A), which indicates that HO^●^ seems to be harmless towards Hfra. Therefore, not even the tiny amount of HO^●^ formed in presence of Hfra (Figure 4B) is able to damage this protein. Consequently, our results prove that Hfra is not oxidized under the conditions used to mimic the cellular oxidative stress. This occurs because Hfra has a notorious potential to inhibit the formation of free radicals, but also because it seems to be somehow resistant to the HO^●^–induced damage.

The intervention of Yfh1 and Hfra against reactions stimulating the cellular oxidative stress could have involved their self-oxidation, which likely would have had consequences on their biological functions. However, our data conclusively prove that Yfh1 and Hfra are able to reduce the free radical formation without undergoing self-oxidation. Therefore, these results strengthen their biological role as antioxidants even further.

### 3.7. Hfra and Yfh1 Are Able to Protect a-Syn Against Oxidation

Here, we have proven that Hfra and Yfh1 might be directly integrated within the mitochondrial and cytoplasmic antioxidant machineries. If this occurs, it would be through their ability to reduce the ROS release, which indirectly involves their self-protection against oxidation. From these observations we wonder whether frataxins could also protect other intracellular proteins susceptible to oxidative modifications.

This study was carried out using α-syn, an intrinsically disorded protein mainly found in the cytoplasm of the neurons of the substantia nigra [72]. There, α-syn plays a broad set of physiological roles, among which highlights its ability to regulate the neurotransmission. However, α-syn is also well-known to aggregate through the formation of intraneuronal deposits, known as Lewy bodies (LBs), which are responsible for the development of Parkinson’s disease [73]. The formation of LBs is stimulated by α-syn mutations [74]; the formation of α-syn-metal complexes [75]; or the oxidation of α-syn [76,77], which initiates the feed-forward cycle of oxidized α-syn-induced stress detected in Parkinson’s disease [78]. Here, we selected α-syn as a plausible protecting target of frataxin from the results obtained by Kim et al., who proved that a Tat-fused Hfra reduced the oxidative stress in dopaminergic neurons [43]. This indicates that frataxins could protect α-syn against oxidative stress and reduce the propensity to develop Parkinson’s disease.

**Figure 6:**
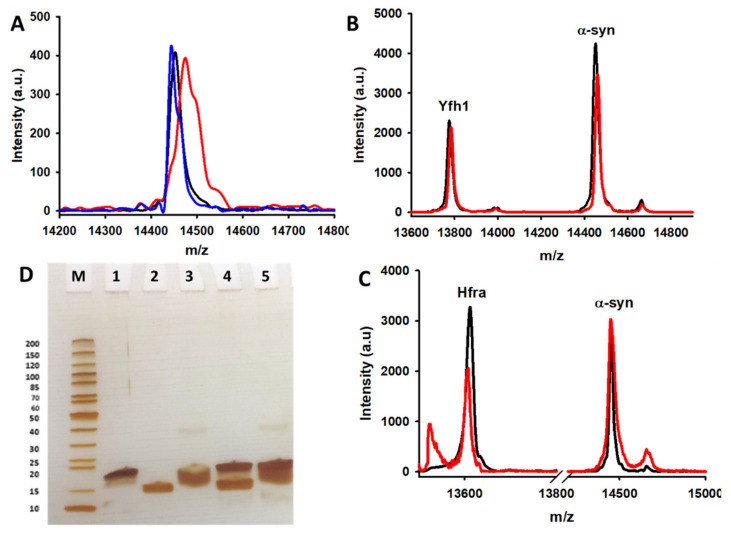
Studying the protective role of Yfh1 and Hfra on oxidation of α-syn. (**A**) MALDI-TOF/TOF signal of α-syn (10 μM) incubated during 0 min (*black*) and 150 min (*blue and red*) in the presence of AA (70 μM) (*blue*) or in the presence of AA (70 μM) and Cu^2+^ (2.5 μM) (*red*). (**B**) MALDI-TOF/TOF signals of Yfh1 (5 μM) and α-syn (10 μM) co-incubated with AA (70 μM) and Cu^2+^ (2.5 μM) during 0 min (*black*) and 150 min (*red*). (**C**) MALDI-TOF/TOF signals of Hfra (5 μM) and α-syn (10 μM) co-incubated with AA (70 μM) and Cu^2+^ (2.5 μM) during 0 min (*black*) and 150 min (*red*). (**D**) SDS-PAGE electrophoretic gel of the analysis of reaction mixtures containing 0.1mM EGS and: (1) 10 μM α-syn; (2) 10 μM Hfra; (3) 10 μM Yfh1; (4) 10 μM α-syn and 10 μM Hfra; and (5) 10 μM α-syn and 10 μM Yfh1. These mixtures were incubated during 30 min at 25 °C before analysis. The gel includes a marker (M) where each protein band has been labelled with its molecular weight (*left*).

We have previously shown that the affinity of α-syn towards Cu^2+^ (it has three binding regions with *K*_d_ between 4 and 200 μM), and the formation of the corresponding complexes, slightly reduced the rate of the Cu^2+^-catalyzed AA degradation while diminishing the formation of ROS [46]. However, this does not preclude α-syn of being oxidized during this process, as we clearly observed a remarkable increase in its molecular weight when it was incubated with AA and Cu^2+^ (Figure 6A). Likely, this occurs due to the susceptibility of α-syn to be damaged by HO^●^ (Figure 5B), although the H_2_O_2_ formed during the Fe^3+^-catalyzed AA degradation (Figure 3B)—it is the main ROS formed during this process (Figure S8)—seems to also damage α-syn, as its molecular weight also slightly increased when it was incubated with Fe^3+^ and AA (Figure S11).

Nonetheless, the molecular weight of α-syn did not change when it was incubated with Cu^2+^/Fe^3+^ and AA in the presence of frataxins (Figure 6B,C and Figure S12), even though when the concentrations of Yfh1 or Hfra were two-fold lower. However, the protecting mechanism of Yfh1 and Hfra on α-syn seems to be rather different. The presence of Yfh1 only scarcely protect α-syn to react with chemically synthetized HO^●^ radicals (Figure 5B), thus the protecting ability of Yfh1 must be due to its intrinsic ability to inhibit the metal-catalyzed degradation of AA and the formation of ROS able to damage α-syn. In contrast, the presence of Hfra completely protected α-syn against free HO^●^ radicals (Figure 5B). Hence, although Hfra is not able to inhibit the metal-catalyzed degradation of AA, it is able to direct its oxidation towards the formation of species different than ROS, but at the same time it is able to protect α-syn against oxidative stress.

The obtained results prove that both frataxins are capable to protect other cytoplasmatic proteins against the oxidation induced by an unbalanced oxidative stress. In addition, our data strengthen the idea suggested by Kim et al. that frataxins can be pharmacologically used against the development of Parkinson’s disease as they can avoid α-syn oxidation and therefore, the formation of LBs.

### 3.8. Protective Effect of Hfra and Yfh1 on a-Syn Does Not Involve Their Direct Binding

The obtained data brought an intriguing question that is to know whether the presence per se of frataxins is enough to protect α-syn or on the contrary, the protecting mechanism implies the formation of protein complexes between Yfh1/Hfra and α-syn. To investigate if frataxins were able to bind α-syn, we performed chemical cross-linking experiments using EGS, a 16-Ǻ linear molecule that forms covalent links between “close enough” amino groups (α-syn has 15 Lys residues in its sequence) [79]. SDS-PAGE analysis of cross-linked samples did not show the appearance of any band ascribable to a frataxin-α-syn complex (Figure 6D). Moreover, MALDI-TOF/TOF data also points towards the same direction. We observed several MALDI peaks in the theoretical dimeric region, but their molecular weights correspond to Yfh1–Yfh1, Hfra–Hfra, and α-syn–α-syn homodimers and in any case, they were also formed in the absence of EGS. We could not detect any MALDI peak corresponding to the Yfh1-α-syn (~28.1 kDa) or the Hfra-α-syn (~27.9 kDa) heterodimers (Figure S13A,B).

Hence, these results indicate that frataxins do not interact with α-syn time enough to be cross-linked, thus it is highly unlikely that they form stable complexes. Consequently, the observed protecting effect of frataxins on α-syn do not involve their binding and the solely presence of frataxins seems to be enough to avoid α-syn oxidation. Obviously, this is an unspecific effect, which proves that frataxins might be able to protect a broad set of cytoplasmatic molecules against the oxidative stress.

## 4. Conclusions

FRDA is a rare neurodegenerative disease caused by a deficiency in the expression levels of frataxin, a small mitochondrial protein considered as an iron-binding protein. In fact, the biological functions of frataxin are intimately related with the iron metabolism. Frataxin is able to store iron and then, transfer it to other proteins. Moreover, frataxin seems to be directly involved in the biosynthesis of the heme groups and of the Fe-S clusters. In addition, it has been suggested that frataxins (Yfh1 and Hfra but not CyaY) have a crucial role protecting cells against oxidative stress. To date, this biological function was attributed to their ability to regulate the enzymatic antioxidant defenses. However, the metal binding affinities shown by frataxins let us to hypothesize that they could directly act as part of the cellular antioxidant defense.

Here, we have proved that Yfh1 completely inhibits the Cu^2+^- and the Fe^3+^-catalyzed degradation of AA. Therefore, Yfh1 is also able to diminish the formation of H_2_O_2_ that would appear from the O_2_ and the O_2_^●−^ sequential reductions and consequently, the formation of free radicals such as HO^●^. Although Yfh1 can be easily oxidized by HO^●^, its antioxidant activity does not imply its self-oxidation basically because it does prevent the metal-catalyzed oxidations yielding ROS.

On the other hand, Hfra speeds up the Cu^2+^-catalyzed degradation of AA towards the formation of remarkable amounts of H_2_O_2_. Similarly, Hfra also accelerates the Fe^3+^-catalyzed degradation of AA but redirects this process towards the formation of other compounds different than H_2_O_2_, which likely do not contribute to the cellular oxidative stress. In any case, Hfra is able to considerably reduce the formation of free radicals (mainly HO^●^) arising from both, the Cu^2+^- and the Fe^3+^-catalyzed degradation of AA. The tiny amounts of HO^●^ formed from these processes are unable to oxidize Hfra, which antioxidant intervention does not imply its self-oxidation.

Consequently, our work proves that Yfh1 and Hfra are essential components of the intracellular antioxidant machinery, not only because they are able to regulate the detoxifying enzymatic mechanisms, but also because they directly act against the production of ROS. This latter mechanism also has further consequences since both proteins are able to unspecifically inhibit the oxidation of α-syn, which prove that they could act as molecular shields to protect a broad set of intracellular oxidation-prone proteins.

## Data Availability

Data is contained within the article or Supplementary Materials.

## Supplementary Materials

The following are available online at https://www.mdpi.com/2076-3921/10/2/315/s1, Figure S1: Structural and sequential comparisons between bacterial, yeast and human frataxins; Figure S2: Representation of the hydrophobic and negatively charged residues on the tridimensional structures of frataxin; Figure S3: Effect of NaCl and phosphate concentration on the degradation rate of AA; Figure S4: Effect of Cu^2+^, Fe^3+^, EDTA, and O_2_ on the degradation rate of AA; Figure S5: Time-dependent AA degradation in the absence of metal cations and in the presence of Yfh1 and Hfra; Figure S6: DLS studies on the Cu^2+^- and Fe^3+^-induced oligomerization of Hfra; Figure S7: UV–vis spectroscopic study of the formation of O_2_^●−^ radical in the presence of AA; Figure S8: Effect Fe^3+^ on the overall free radical and hydroxyl radical (OH^●^) formation from the AA degradation; Figure S9: Evaluating the oxidation of frataxins during their incubation with metals and AA; Figure S10: Fluorescence study of the di-Tyr formation; Figure S11: MALDI-TOF/TOF study of the oxidation of α-syn in the presence of AA and Fe^3+^; Figure S12: MALDI-TOF/TOF study of the oxidation of α-syn in the presence of AA, Fe^3+^, Hfra and Yfh1; Figure S13: MALDI-TOF/TOF of the complex formation between frataxins and α-syn; Table S1: Stoichiometry and dissociation constants determined for the complexes formed between different frataxins and iron and cooper cations.

## Abbreviations

The following abbreviations are used in this manuscript:

FRDA	Friedreich’s ataxia
CyaY	Bacterial frataxin
Hfra	Human frataxin
Yfh1	Yeast frataxin
ROS	Reactive oxygen species
SODs	Superoxide dismutases
AA	Ascorbic acid
LB	Luria Bertani media
IPTG	Isopropyl-β-D-1-thiogalactopyranoside
α-syn	Human α-synuclein
Buffer B1	10 mM phosphate buffer (pH 7.4) containing 150 mM of NaCl
NBT	Nitroblue tetrazolium
EGS	Ethylene glycol bis-succinimidyl succinate
LBs	Lewy bodies
