# Supplementary material for: Frataxins Emerge as New Players of the Intracellular Antioxidant Machinery
Source: Antioxidants (Basel). 2021 Feb 20;10(2):315. doi: 10.3390/antiox10020315 (PMC7923443; doi:10.3390/antiox10020315)
Supplement: Supplementary file 1 [file antioxidants-10-00315-s001.pdf]

A

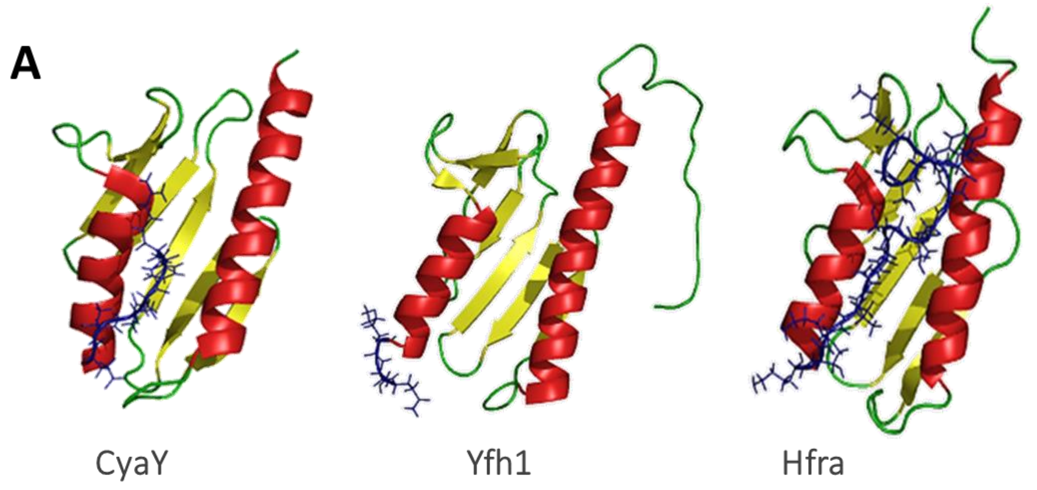

B

```
        1         10        20        30        40    ----   50        60
CYAY  -------------------------------------------------------------------------
YFH1  ---MIKRSLASLV------------------RVSSVMGRRYMIAAAGGERARFCPAVT-NKKNHTVNTF---Q
HFRA  MWTLGRRAVAGLLASPSPAQAQTLTRVPRPAELAPLCGRRGLRTDI----DATCTPRRASSNQRGLNQIWNVK

        70        80      ---90       100       110   -    120       130
CYAY  -----------------------MNDSEFHRLADQLWLTIEERLDDWD-----GDSDIDCEINGGVLTITFE-
YFH1  K--R-FVESSTDGQV-VPQEVLNLPLEKYHEEADDYLDHLLDSLEELSEAHPDCIPD--VELSHGVMTLEI-P
HFRA  KQSVYLMNLRKSGTLGHPGS---LDETTYERLAEETLDSLAEFFEDLAD-KPYTFEDYDVSFGSGVLTVKLGG

         140       150       160       170       180       190       200       210
CYAY  NGSKIIINRQEPLHQVWLATKQ-GGYHFDLKGDEWICDRSGETFWDLLEQAATQQAGETVSFR---------
YFH1  AFGTYVINKQPPNKQIWLASPLSGPNRFDLLNGEWVSLRNGTKLTDILTEEVEKAISKSQ------------
HFRA  DLGTYVINKQTPNKQIWLSSPSSGPKRYDWTGKNWVYSHDGVSLHELLAAELTKALKTKLDLSSLAYSGKDA
```

Figure S1. Structural and sequential comparisons between bacterial, yeast and human frataxins. **(A)** Tridimensional structures of the globular region of bacterial frataxin (CyaY; PDB code 1SOY), yeast frataxin (Yfh1; PDB code 2GA5) and human frataxin (Hfra; PDB code 1LY7). Alpha-helical regions are shown in red; β-sheets are shown in yellow; and in blue is shown the unfolded C-terminal tail, which length varies between the different species. **(B)** Sequential alignment of the mature form of CyaY (Swiss-pro code: P27838), Yfh1 (Swiss-pro code: Q07540) and Hfra (Swiss-pro code: Q16595) carried out using the Clustal Omega software. Residues in green are those maintained in the three sequences. Residues in blue and red are those not conserved but with similar chemical properties.

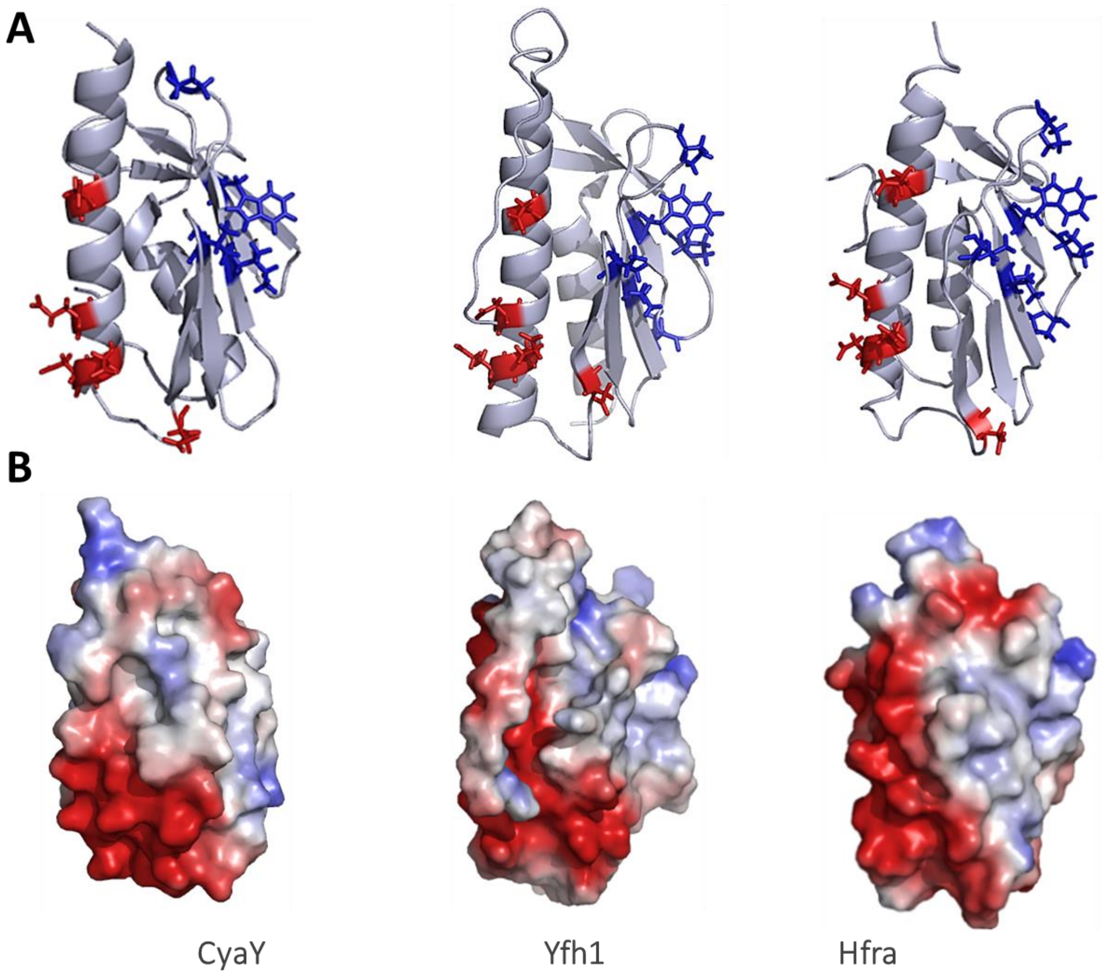

Figure S2. Representation of the hydrophobic and negatively charged residues on the tridimensional structures of frataxin. **(A)** Cartoon representation of the tridimensional structures of the globular region of bacterial (CyaY; PDB code 1SOY), yeast (Yfh1; PDB code 2GA5) and human frataxins (Hfra; PDB code 1LY7). The backbone is shown in grey, whereas the side chains of negatively charged residues (*red*) and hydrophobic residues (*blue*) are shown as sticks. **(B)** Electrostatic surface potential of the CyaY, Yfh1 and Hfra tridimensional structures (the orientation of each structure is the same than that shown in panel A). The colors are fixed from red (-5) to blue (+5).

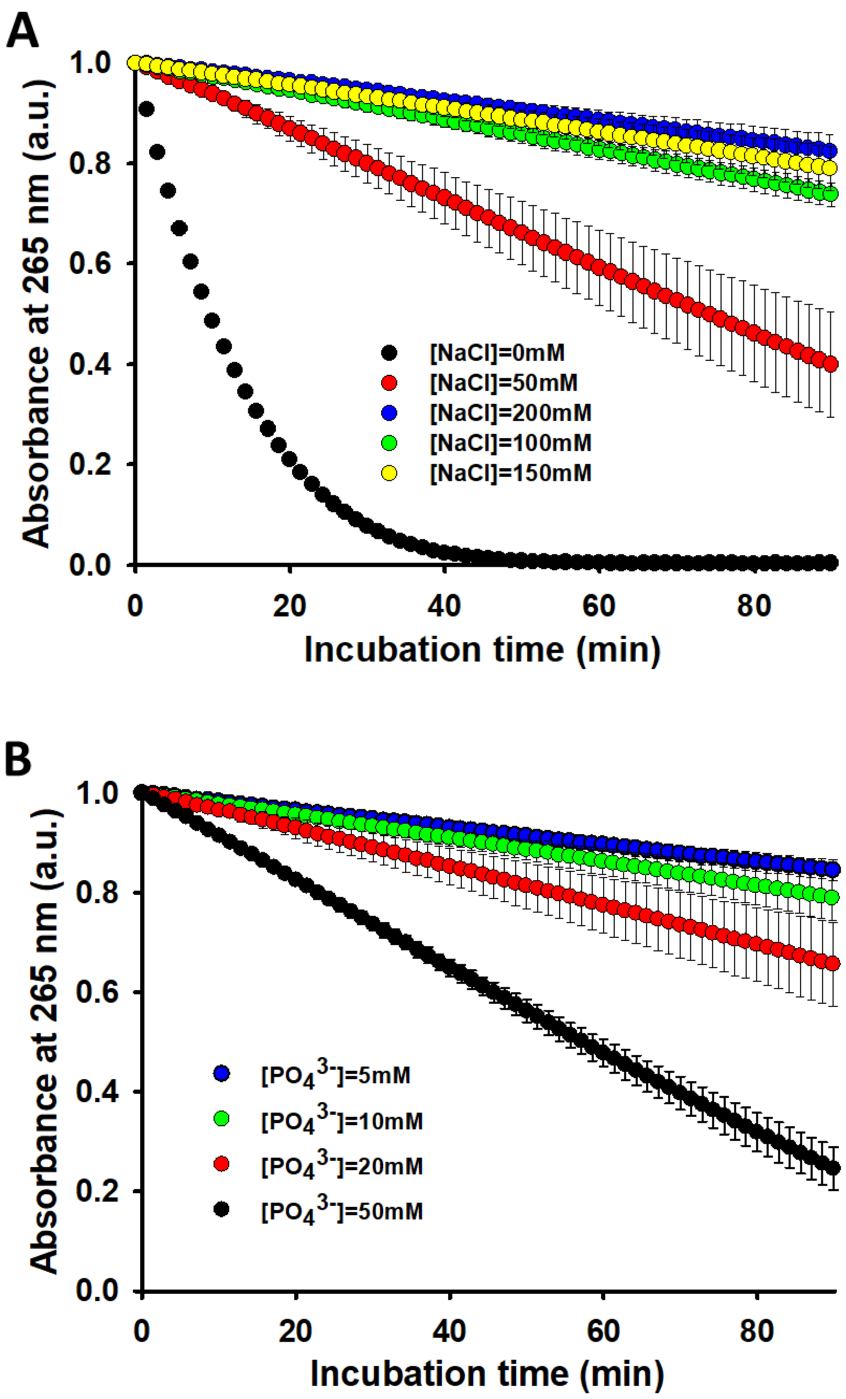

Figure S3. Effect of NaCl and phosphate concentration on the degradation rate of AA. **(A)** Time-dependent AA (70 µM) degradation in 10 mM phosphate buffer (pH 7.4) at 25°C measured by the decrease in its absorbance at 265 nm when AA was alone (●) or in the presence of NaCl at 50 mM (●), 100 mM (●), 150 mM (●) and 200 mM (●) concentration; **(B)** Time-dependent AA (70 µM) degradation at 25°C in the presence of NaCl (150 mM) and in buffers (pH 7.4) containing 5 mM (●), 10 mM (●), 20 mM (●) or 50 mM (●) phosphate concentration. In both panels, the data points are the mean from all experiments, and the error bars represent standard deviation from the different independent measurements.

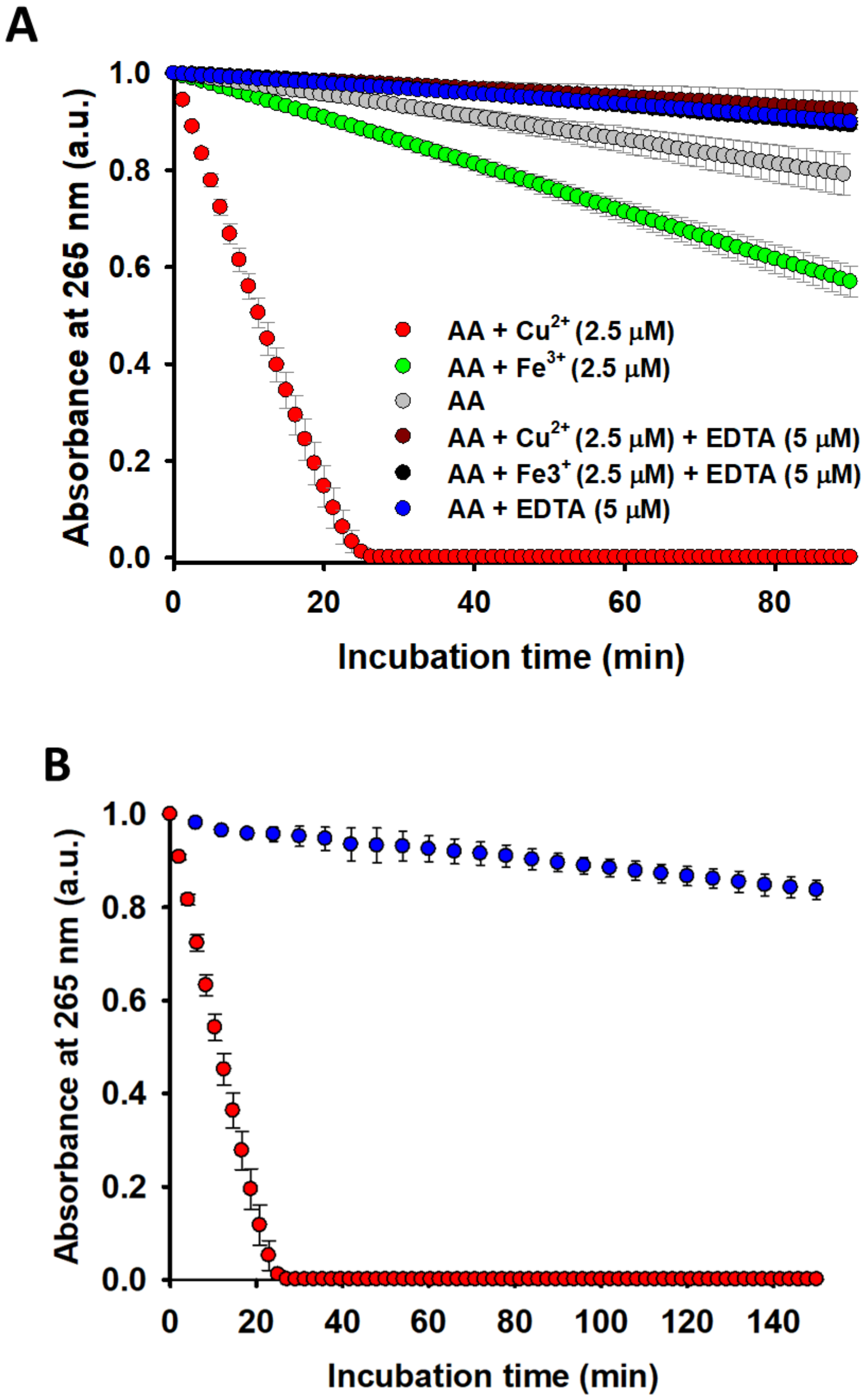

Figure S4. Effect of Cu^{2+}, Fe^{3+}, EDTA and O_2 on the degradation rate of AA. **(A)** Time-dependent AA (70 µM) degradation at 25°C measured by the decrease in its absorbance at 265 nm when AA was alone (●), in presence of Cu^{2+} (2.5 µM) (●), in presence of Fe^{3+} (2.5 µM) (●), in presence of EDTA (5 µM) (●), in presence of Cu^{2+} (2.5 µM) and EDTA (5 µM) (●), and in presence of Fe^{3+} (2.5 µM) and EDTA (5 µM) (●). **(B)** Time-dependent AA (70 µM) degradation in the presence of Cu^{2+} (2.5 µM) using a freshly prepared buffer B1 (●), and a buffer B1 that was previously subjected to N_2 bubbling during 15min to eliminate the molecular oxygen (O_2) dissolved in it (●). In both panels, the data points are the mean from all experiments, and the error bars represent standard deviation from the different independent measurements.

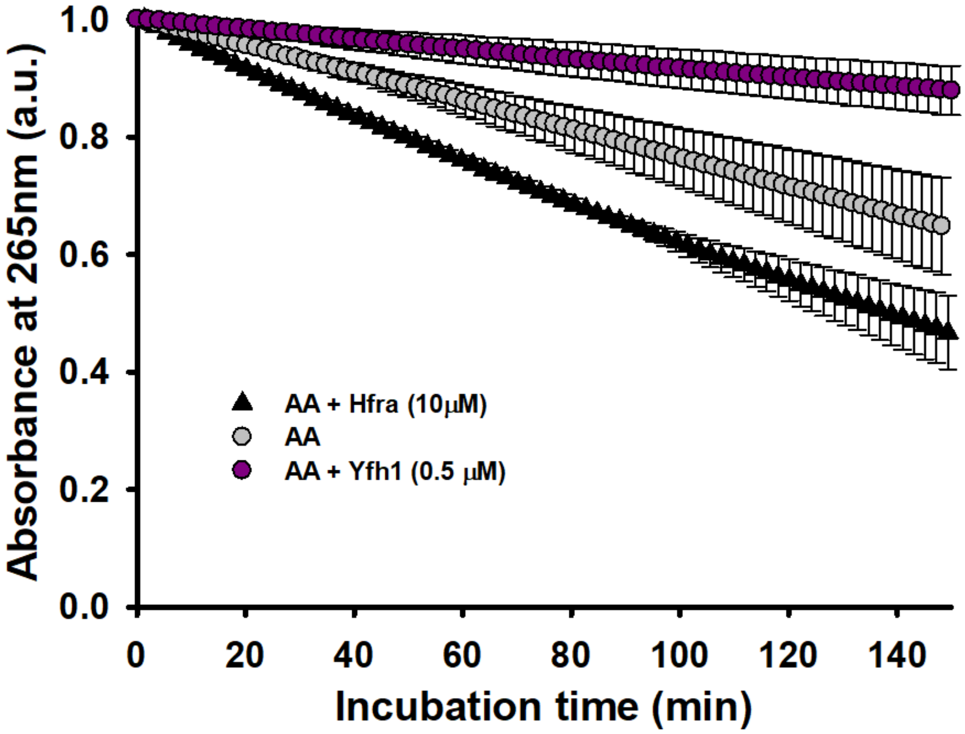

Figure S5. Time-dependent AA (70μM) degradation at 25°C measured by the decrease in its absorbance at 265nm when AA was incubated alone (●), in presence of Yfh1 (0.5 μM) (●), or in the presence of Hfra (10 μM) (▲). In both panels, the data points are the mean from all experiments, and the error bars represent standard deviation from the different independent measurements.

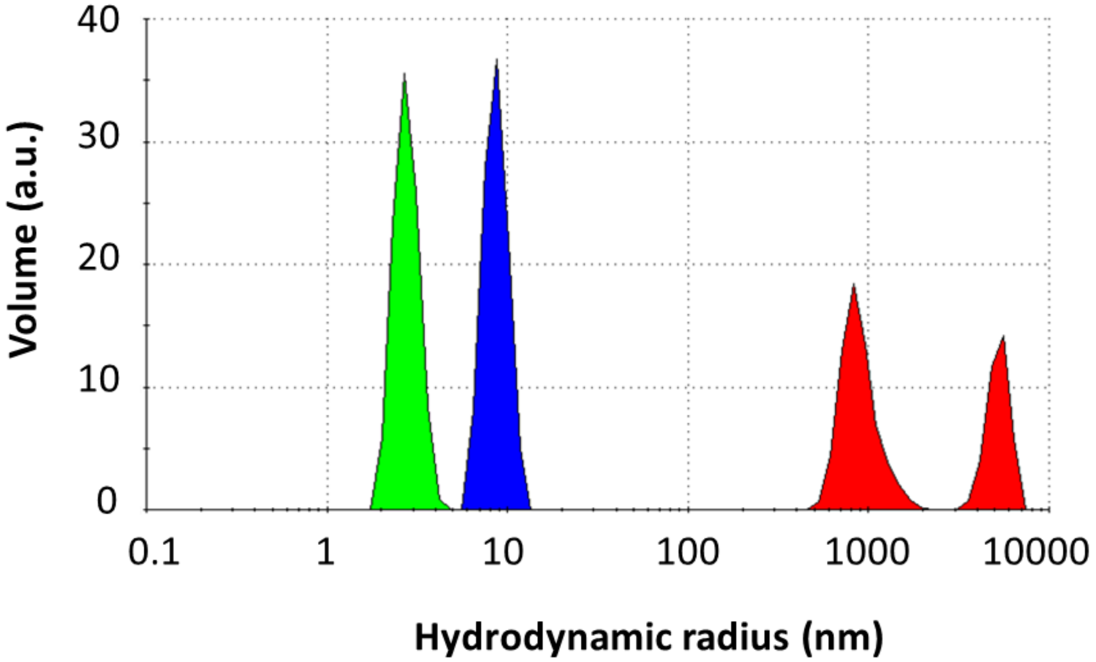

Figure S6. DLS studies on the Cu^{2+}- and Fe^{3+}-induced oligomerization of Hfra. Hfra (60 µM) size was measured alone (green peak), in the presence of Cu^{2+} (0.6 mM; blue peak) and in the presence of Fe^{3+} (0.6 mM; red peaks). The measurements were carried out in buffer B1. The measurements were performed at 25°C just after the metal cations were added to the solutions containing Hfra.

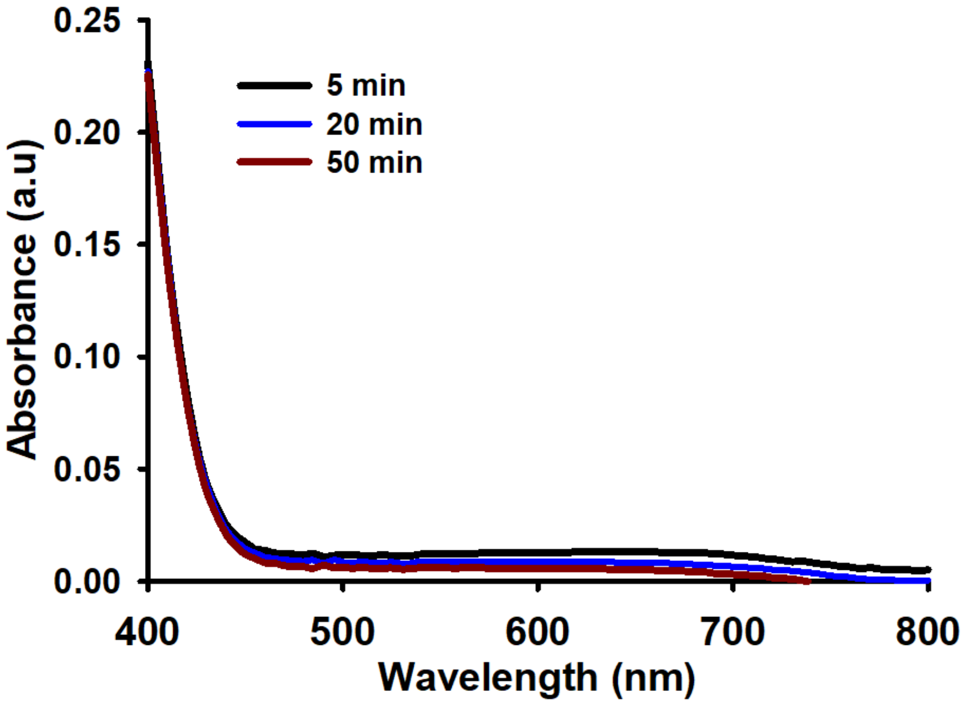

Figure S7. UV-Vis spectra of a reaction mixture containing AA (70 µM), Fe^{3+} (2.5 µM) and NBT (50 µM) in 10 mM sodium phosphate buffer at pH 7.4 after 5 min, 20 min and 50 min of incubation at 25°C. These plots indicate that Fe^{3+}-catalyzed AA degradation does not involve a temporal increase in the free $O_2^{\bullet-}$ concentration.

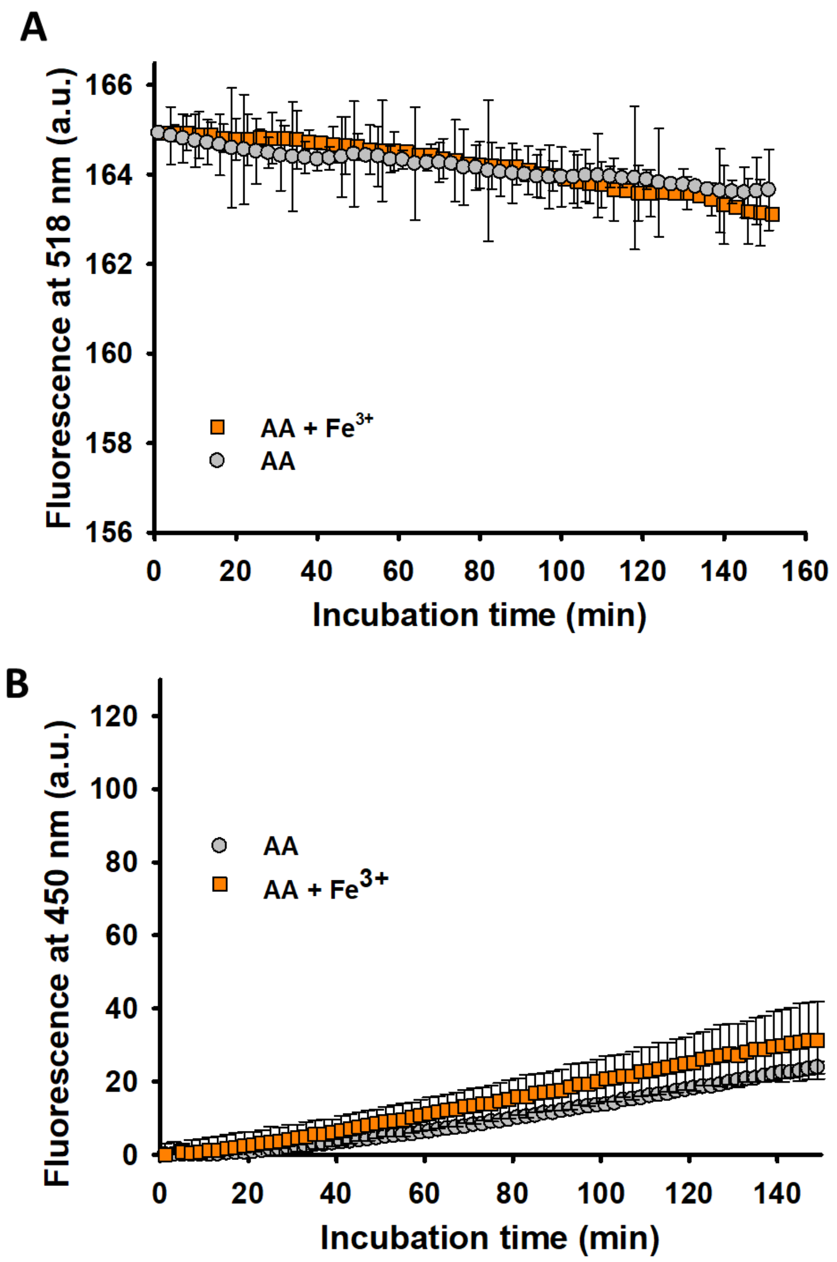

Figure S8. Effect Fe^{3+} on the overall free radical and hydroxyl radical (OH•) formation from the AA degradation. **(A)** Time-dependent overall free radical formation monitored by the decrease in the fluorescence intensity of fluorescein (26 µM; λ_{exc}=490 nm) of a solution prepared in buffer B1 that contained AA (70 µM) alone (●) or in the presence of Fe^{3+} (2.5 µM) (■). The experimental data was smoothed using the negative exponential function in Sigmaplot. **(B)** Time-dependent formation of HO• measured by the increase in the fluorescence of 3-CAA at 450 nm (λ_{exc}=395 nm) of a solution prepared in buffer B1 that contained AA (70 µM) alone (●) or in the presence of Fe^{3+} (2.5 µM) (■). In both panels, the data points are the mean from all experiments, and the error bars represent standard deviation from the different independent measurements.

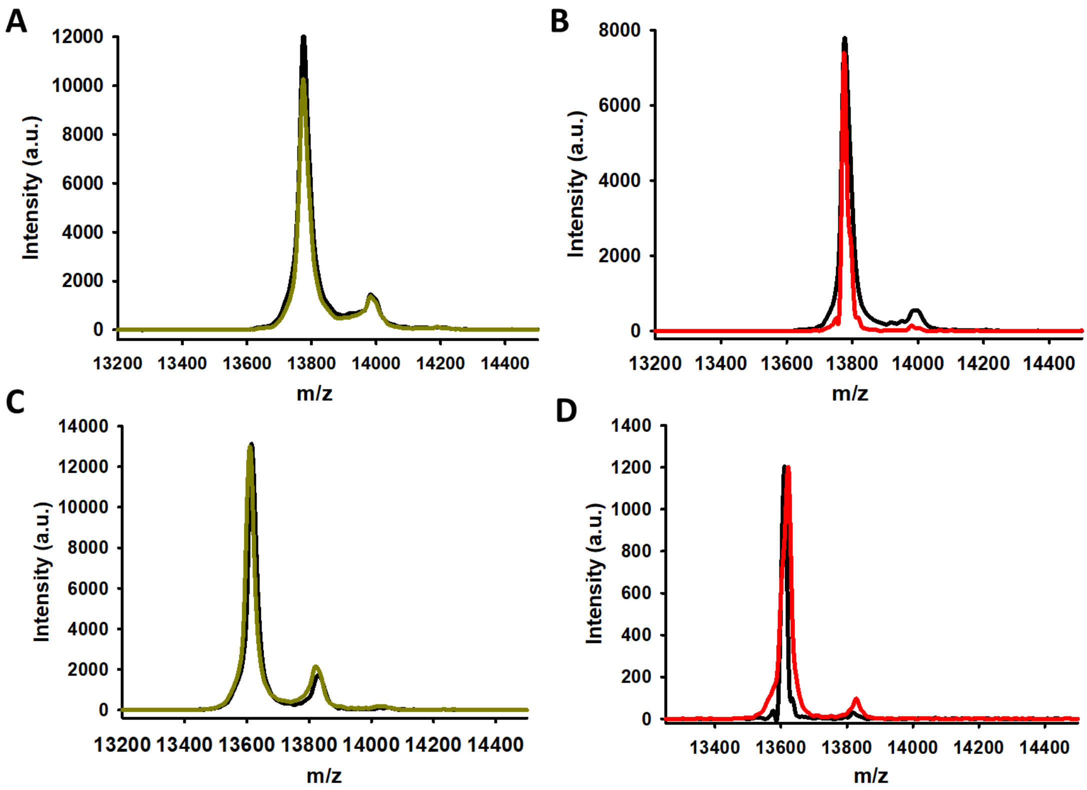

Figure S9. Evaluating the oxidation of frataxins during their incubation with metals and AA. **(A-B)** MALDI-TOF/TOF signal of Yfh1 incubated during 0 min (*black*) and 150 min (*dark-yellow*) at 25 °C in buffer B1 with AA (70 µM) and Fe^{3+} (2.5 µM) **(A)**, or Cu^{2+} (2.5 µM) **(B)**. **(C-D)** MALDI-TOF/TOF signal of Hfra incubated during 0 min (*black*) and 150 min (*dark-yellow*) at 25 °C in buffer B1 with AA (70 µM) and Fe^{3+} (2.5 µM) **(C)**, or Cu^{2+} (2.5 µM) **(D)**.

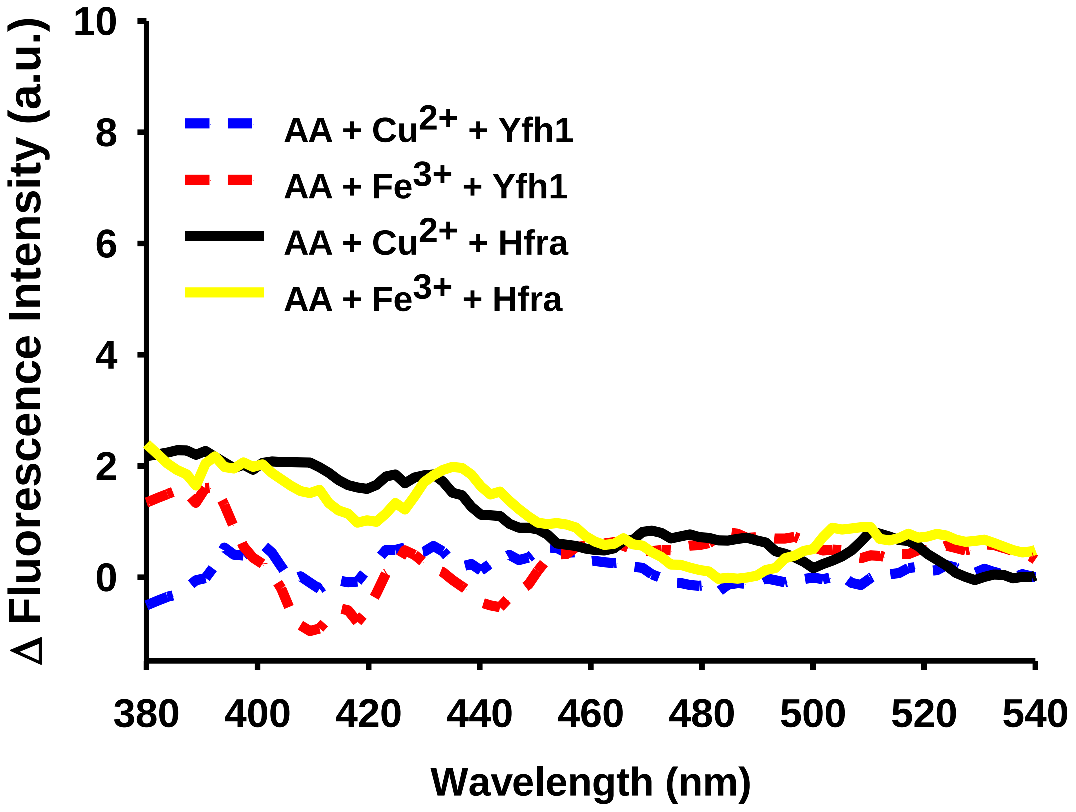

Figure S10. Differences between the fluorescence spectra of Yfh1 (10 µM) (*dashed lines*) and Hfra (10 µM) (*lines*) before and after 150 min incubation in the presence of 70 µM AA and 2.5 µM of Cu^{2+} or Fe^{3+}.The experimental data was smoothed using the negative exponential function in Sigmaplot. These experiments were carried out in duplicate (n=2).

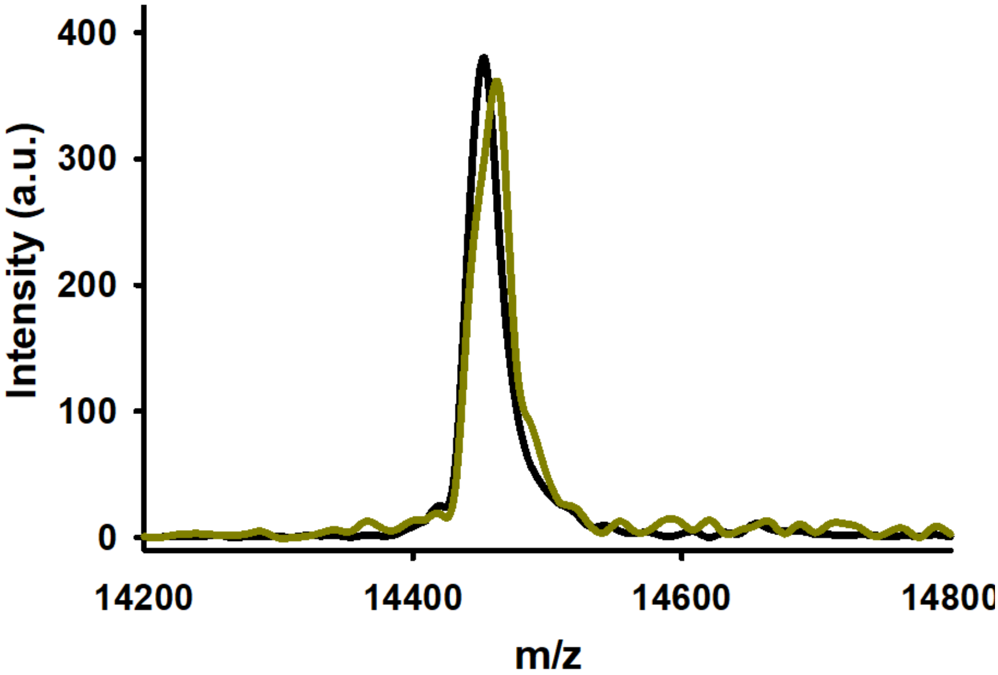

Figure S11. MALDI-TOF/TOF signal of α-syn (10 µM) incubated during 0 min (*black*) and 150 min in the presence of AA (70 µM) and Fe^{3+} (2.5 µM) (*dark-yellow*).

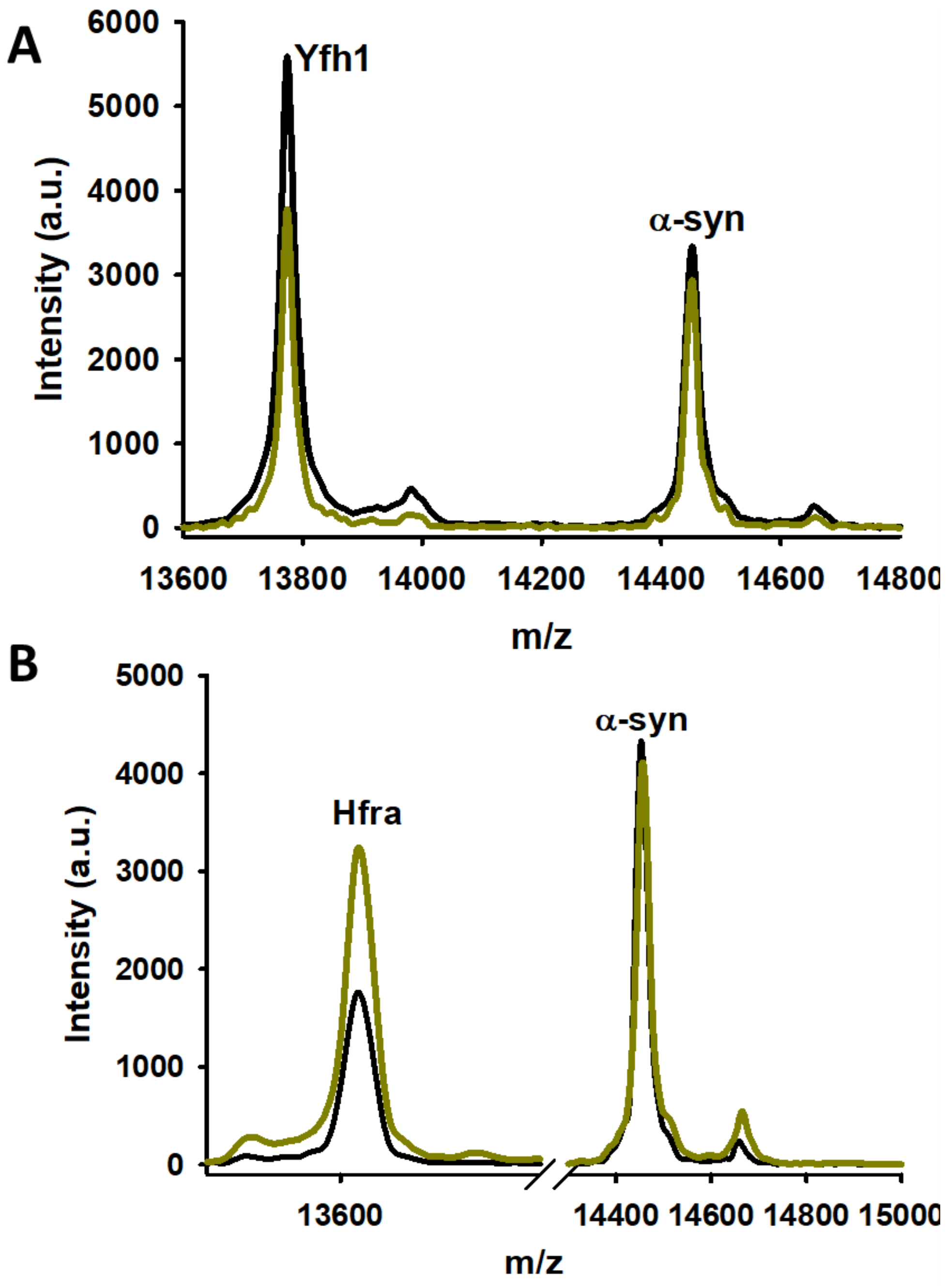

Figure S12. MALDI-TOF/TOF study of the oxidation of α-syn in presence of frataxins during their incubation with AA and Fe^{3+}. **(A)** MALDI-TOF/TOF signals of Yfh1 (5 µM) and α-syn (10 µM) co-incubated during 0 min (*black*) and 150 min (*dark-yellow*) with AA (70 µM) and Fe^{3+} (2.5 µM). **(B)** MALDI-TOF/TOF signals of Hfra (5 µM) and α-syn (10 µM) co-incubated during 0 min (*black*) and 150 min (*dark-yellow*) with AA (70 µM) and Fe^{3+} (2.5 µM).

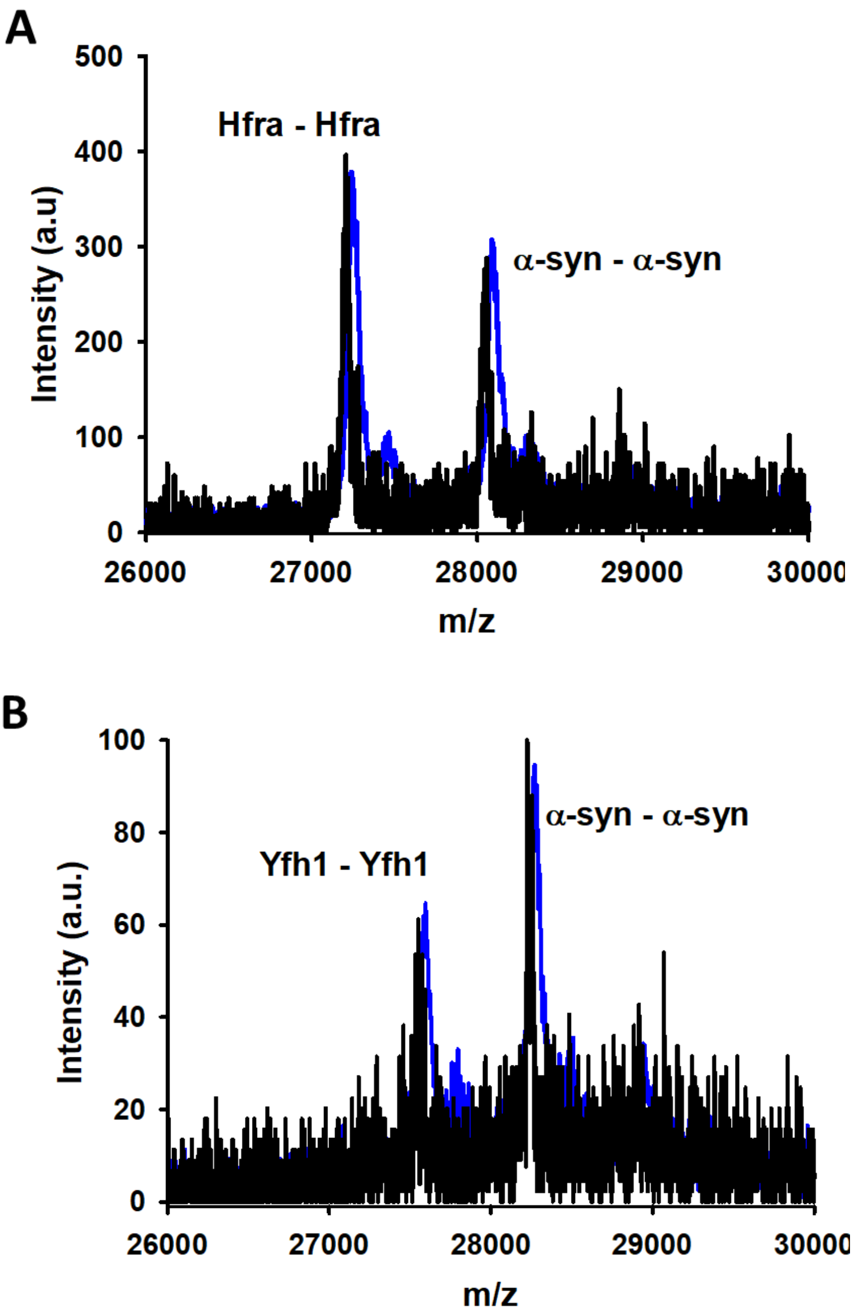

Figure S13. Study of the formation of protein-protein complexes between Hfra or Yfh1 with α-syn. **(A)** MALDI-TOF/TOF spectra of a reaction mixture containing Hfra (10 µM) and α-syn (10 µM) incubated during 30 min at 25°C in the absence (*black*) and in the presence of 0.1 mM EGS (*blue*). The peaks corresponding to the Hfra-Hfra and α-syn-α-syn homodimers are labelled. **(B)** MALDI-TOF/TOF spectra of a reaction mixture containing Yfh1 (10 µM) and α-syn (10 µM) incubated during 30 min at 25°C in the absence (*black*) and in the presence of 0.1 mM EGS (*blue*). The peaks corresponding to the Yfh1-Yfh1 and α-syn-α-syn homodimers are labelled

Table S1. Stoichiometry (*n*) and dissociation constants (K_d) determined for the complexes formed between different frataxins and iron and cooper cations.

Protein	Cation	*n*	K_d (µM)	Reference
CyaY		1:2	3.80[b]	[1]
Yfh1	Fe^{2+}	1:2[a]	3.00 / 2.00	[2]
Hfra		1:7	55.0[b]	[3]
CyaY		1:7[a]	-	[1]
Yfh1	Fe^{3+}	1:1	29.4	[4]
Hfra		1:7	11.0[b]	[3]
Yfh1	Cu^{+}	1:1	0.032	[4]
Yfh1	Cu^{2+}	1:2	0.13 / 310	[4]

a) This stoichiometry only includes the higher affinity binding regions, although it is described that CyaY and Yfh1 are able to bind, with a low affinity, a higher number of Fe^{3+} and Fe^{2+} cations, respectively.

b) This dissociation constant shows an apparent dissociation constant that encompass all the binding regions of the metal cation to the protein.

REFERENCES

1. Bou-Abdallah, F.; Adinolfi, S.; Pastore, A.; Laue, T. M.; Chasteen, N. D. Iron binding and oxidation kinetics in frataxin CyaY of escherichia coli. *J. Mol. Biol.* **2004**, 341, 605-615.
2. Cook, J.; Bencze, K.; Jankovic, A.; Crater, A. K.; Busch, C. N.; Bradley, P. B.;Stemmler, A. J.; Spaller, M. R.; Stemmler T. L. Monomeric yeast frataxin is an iron binding protein. *Biochemistry*, **2006**, 45, 7767–7777.
3. Yoon, T.; Cowan, J. Iron-sulfur cluster biosynthesis. Characterization of frataxin as an iron donor for assembly of [2Fe-2S] clusters in ISU-type proteins. *J. Am. Chem. Soc.*, **2003**, 125, 6078–6084
4. Han, T.; Camadro, J.; Santos, R.; Lesuisse, E.; El Hage Chahine, J. M.; Ha-Duong, N. T. Mechanisms of iron and copper-frataxin interactions. *Metallomics*, **2017**, 9, 1073–1085.
